# Metformin induces S‐adenosylmethionine restriction to extend the *Caenorhabditis elegans* healthspan through H3K4me3 modifiers

**DOI:** 10.1111/acel.13567

**Published:** 2022-02-11

**Authors:** Yi Xiao, Fang Liu, Qinghong Kong, Xinting Zhu, Haijuan Wang, Sanhua Li, Nian Jiang, Changyan Yu, Liu Yun

**Affiliations:** ^1^ Institute of life sciences Zunyi Medical University Zunyi China; ^2^ Guizhou Provincial College‐based Key Lab for Tumor Prevention and Treatment with Distinctive Medicines Zunyi Medical University Zunyi China; ^3^ College of Basic Medicine Zunyi Medical University Zunyi China

**Keywords:** *Caenorhabditis elegans*, histone methylation, lifespan, Metformin, mTOR signaling

## Abstract

Metformin, a widely prescribed first‐line drug for the treatment of type II diabetes mellitus, has been shown to extend lifespan and delay the onset of age‐related diseases. The precisely mechanisms by which these effects are realized remain elusive. We find that metformin exposure is restricted to adults, which is sufficient to extend lifespan. However, limiting metformin exposure to the larvae has no significant effect on *Caenorhabditis elegans* longevity. Here, we show that after metformin treatment, the level of S‐adenosylmethionine (SAM) is reduced in adults but not in the larvae. Potential mechanisms by which reduced SAM might increase lifespan include altering the histone methylation. However, the molecular connections between metformin, SAM limitation, methyltransferases, and healthspan‐associated phenotypes are unclear. Through genetic screening of *C*. *elegans*, we find that metformin promotes the healthspan through an H3K4 methyltransferase/demethylase complex to downregulate the targets, including mTOR and S6 kinase. Thus, our studies provide molecular links between meformin, SAM limitation, histone methylation, and healthspan and elucidate the mode action of metformin‐regulated healthspan extension will boost its therapeutic application in the treatment of human aging and age‐related diseases.

## Graphical abstract

Our findings demonstrate that metformin‐induced SAM restriction promotes the healthspan in adults but not in the larvae through an H3K4 methyltransferase/demethylase complex to downregulate the germline targets, including mTOR and S6 kinase.

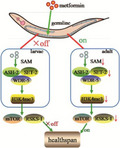

## 1 INTRODUCTION

Metformin, an antiglycemic biguanide drug and the first‐line drug for the treatment of type 2 diabetes, has been proven to extend the lifespan of *Caenorhabditis elegans* (Cabreiro et al., 2013; Chen et al., 2017; De Haes et al., 2014; Onken & Driscoll, 2010; Wu et al., 2016) and mice (Martin‐Montalvo et al., 2013). Because of the wide health benefits and few side effects of metformin, TAME (Targeting Aging with Metformin), a clinical trial, was established to evaluate the protective effects of metformin against human aging and age‐related diseases (Barzilai et al., 2016). Generally, the mechanism of action of metformin, at least in part, depends on AMP‐activated protein kinase (AMPK), a cellular energy sensor (Fryer et al., 2002; Hawley et al., 2002; Zhou et al., 2001), which is involved in energy homeostasis functions, such as glucose and fat metabolism (Larsson et al., 2012). Several studies have shown that metformin may block complex I of the electron transport chain (ETC), leading to an increase in the AMP/ATP ratio and ultimately activating AMPK (Ota et al., 2009; Owen et al., 2000), but this theory is controversial (Larsen et al., 2012; Ouyang et al., 2011). In addition, recent studies suggest that metformin promotes longevity through the Nrf protein ortholog SKN‐1 (Cabreiro et al., 2013; Onken & Driscoll, 2010), the liver kinase B1 ortholog PAR‐4, the AMPK ortholog AMP‐activated kinase‐2 (AAK‐2) (Cabreiro et al., 2013; Onken & Driscoll, 2010), ROS‐peroxiredoxin‐2 (PRDX‐2)‐dependent manner (De Haes et al., 2014), mTORC1 (Wu et al., 2016), and lysosomal pathway (Chen et al., 2017). However, despite its intriguing benefits in promoting healthy aging, the actual mode of action of metformin is largely unknown and a subject of much debate. Overall, many gaps remain in our knowledge of metformin‐induced lifespan extension.

Notably, Cabreiro et al found that metformin increases lifespan at least in part via the AMPK‐activating effects of reduced SAM levels. This suggests that there may be other mechanisms that mediate metformin's induction of SAM restriction to extend lifespan. SAM produced by the 1‐carbon cycle (1CC) is the donor for methylation of histones (Ding et al., 2015). SAM fluctuation may be linked to variations in histone methylation. Low SAM is strongly associated with metabolic disorders, such as fatty liver disease, which is characterized by lipid accumulation, tissue injury, and immune responses (Ding et al., 2015; Halsted et al., 2002; Lu et al., 2001). Furthermore, in mammalian cells, there are more than 200 genes that are predicted to encode SAM‐dependent methyltransferases (Petrossian & Clarke, 2011). However, the mechanisms connecting metformin‐induced SAM restriction to specific methyltransferases and molecular changes underlying healthspan are unknown.

The SAM/SAH ratio imbalance alters histone methylation. Yeast *cho2Δ* mutant cells, which have increased SAM/SAH ratios, have significantly increased trimethylation marks, namely, H3K4me3, H3K36me3, and H3K79me3 (Parkhitko et al., 2019; Ye et al., 2017). Liu et al. found that the reduction in SAM via the downregulation of *Sams* led to decreased levels of H3K4me3 and H3K9me2 in *Drosophila* S2 cells (Liu et al., 2015). In addition, threonine‐regulated SAM specifically affects H3K4me3 levels in mouse embryonic stem cells (Shyh‐Chang et al., 2013). Furthermore, in *C*. *elegans*, the decrease in SAM leads to a large decrease in global H3K4me3 levels (Ding et al., 2015). Greer et al. found that knockdown of the members of the ASH‐2 trithorax complex (ASH‐2, WDR‐5.1, and SET‐2), which trimethylates histone H3 at lysine 4 (H3K4), extended the lifespan of *C*. *elegans*. Conversely, knockdown of RBR‐2 (H3K4me3 demethylase) reduced *C*. *elegans* lifespan (Greer et al., 2010). In addition, the Drosophila homologue of RBR‐2, Lid, plays the same role in regulating lifespan in male Drosophila flies that it does in *C*. *elegans* suggesting that these are conserved regulators of lifespan (Li et al., 2010). Interestingly, H3K4 methylation might be a potential mechanistic target for SAM restriction, as this is among the most sensitive histone methylation sites that suppresses SAM restriction (Mentch et al., 2015; Parkhitko et al., 2019). *Caenorhabditis elegans* has been developed as a valuable genetic model for research on the efficacy of metformin in promoting healthspan (Burkewitz et al., 2014). Overall, we sought to determine whether metformin induces S‐adenosylmethionine restriction to extend the *C*. *elegans* healthspan through H3K4me3 modifiers.

In this study, we show that metformin can prolong the adult lifespan of nematodes but not the larval lifespan. Furthermore, our results reveal the mechanisms connecting metformin‐induced SAM restriction to specific methyltransferases and molecular changes underlying healthspan. These findings revealed that the metformin properties related to healthspan may significantly boost its application to improve the patients with age‐related diseases.

## 2 RESULTS

### 2.1 Metformin extends the lifespan and induces SAM restriction in adult worms but not larval worms

**FIGURE 1:**
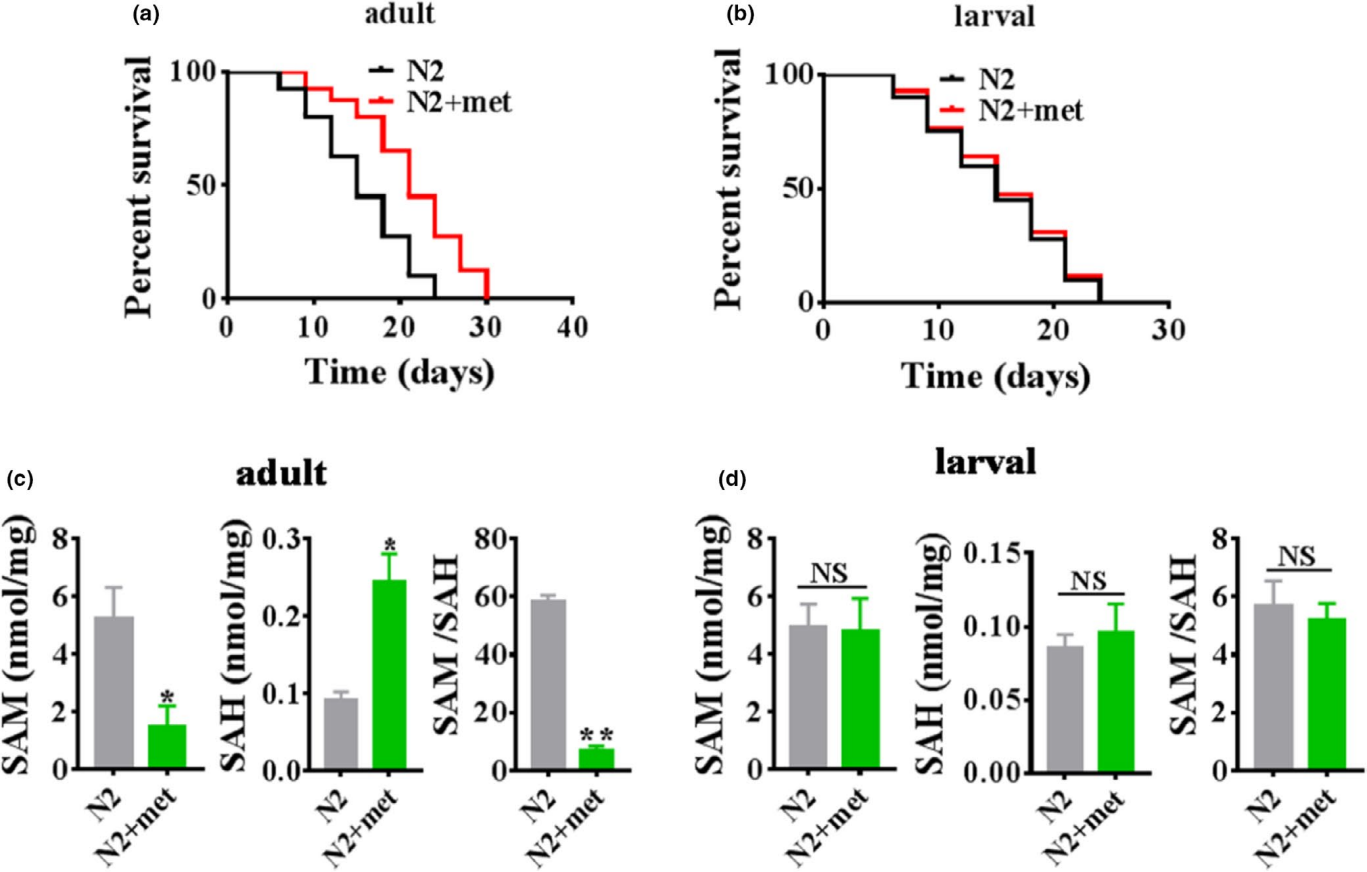
Metformin extends lifespan and induces SAM restriction in adult worms rather than larval worms. (a, b) Metformin (50 mM) promoted longevity in adult worms (a) but not in larval worms (b) (*p < 0.05, log‐rank test). (c, d) The levels of SAM, SAH and SAM/SAH ratio in adult (c) or larval (d) worms exposed to metformin (50 mM) for 24 h. These results are presented as the mean ± SD of three independent experiments performed in triplicate. *p < 0.05 versus N2 (unpaired *t*‐test). NS, no significance

To gain a deeper understanding of the healthspan elicited by metformin exposure, we performed lifespan assays with larval and adult worms. Our results showed that treated with metformin (50 mM) exhibited an increased the lifespan of wild‐type adult worms compared with larval worms (Figure 1a,b, Table S1), consistent with the previous work (De Haes et al., 2014). Precisely how this increase is achieved remains unknown. Previous work has shown that metformin promotes longevity via SAM limitation in *C*. *elegans* (Cabreiro et al., 2013). Notably, metformin (50 mM) treatment decreased the SAM levels, increased the SAH levels, and decreased the SAM/SAH ratio in adult worms but not larval worms (Figure 1c,d). Overall, these results suggest that metformin extends lifespan and induces SAM restriction in adult worms but not larval worms.

### 2.2 SAM restriction induced by metformin inhibits H3K4me3 modifiers

**FIGURE 2:**
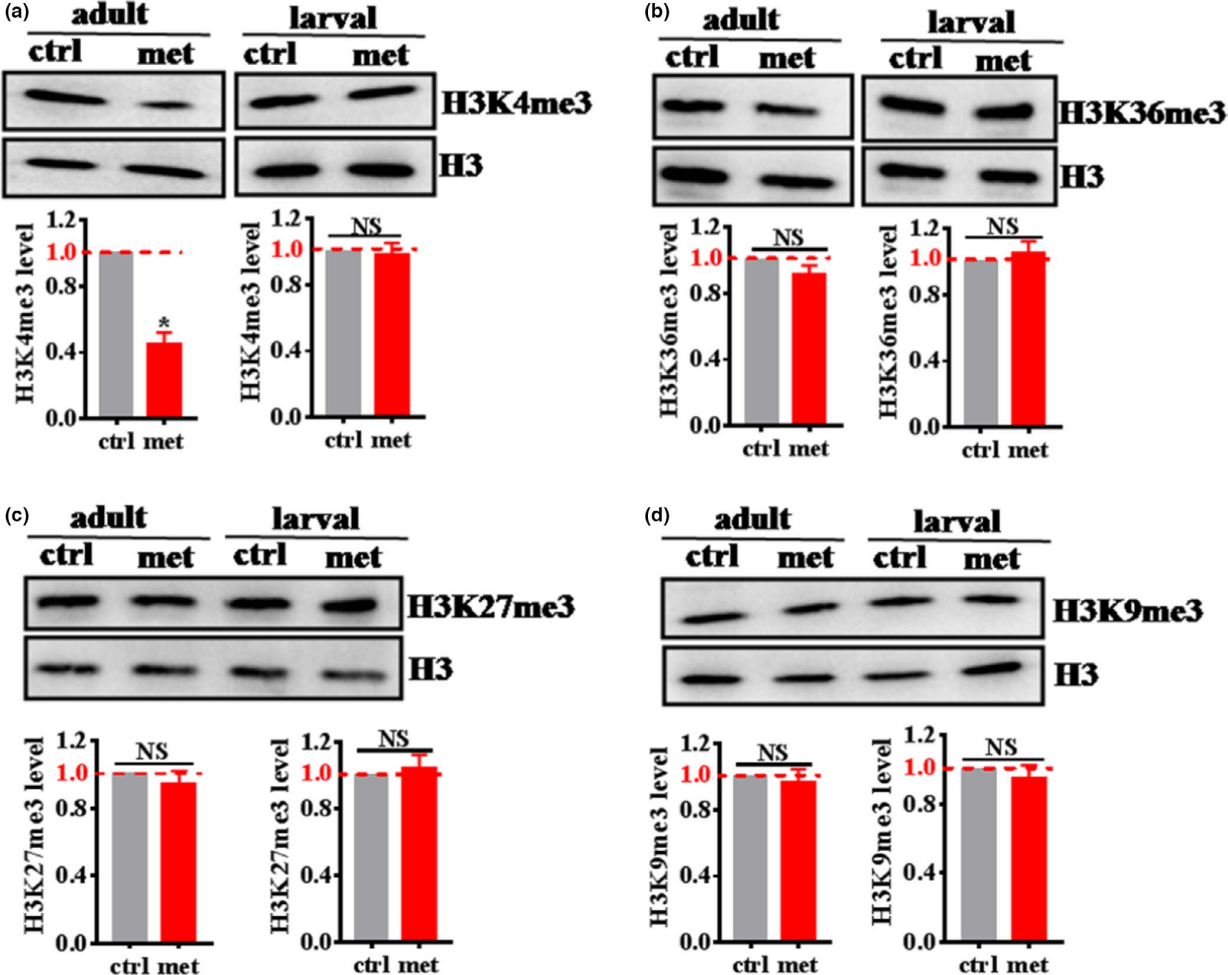
SAM restriction induced by metformin inhibits H3K4me3 modifiers. (a) Metformin significantly decreased the levels of H3K4me3 not in larval worms but in adult worms. After metformin treatment, the levels of H3K36me3 (b), H3K27me3 (c), and H3K9me3 (d) did not change in either adult worms or larval worms. The lower panel shows quantification of H3K4me3 (a), H3K36me3 (b), H3K27me3 (c), and H3K9me3 (d) levels. These results are presented as the mean ± SD of three independent experiments performed in triplicate.*p < 0.05 versus control (unpaired *t*‐test). NS, no significance

SAM is the sole methyl donor modifying histones. Its fluctuation may be linked to variations in histone methylation. Furthermore, in *C*. *elegans*, the decrease in SAM leads to a wide decrease in global H3K4me3 levels (Ding et al., 2015). To determine whether metformin‐induced SAM restriction affected histone trimethylation, we tested four histone trimethylation mark, H3K4me3, H3K9me3, H3K27me3, and H3K36me3 via western blot experiments in adult worms and larval worms. We found that metformin significantly decreased the levels of H3K4me3 not in larval worms but in adult worms (Figure 2a). However, after metformin treatment, the levels of H3K9me3, H3K27me3, and H3K36me3 did not change in either the adult worms or larval worms (Figure 2b–d). Taken together, these results suggest that metformin‐induced SAM restriction affected H3K4me3 modifiers in *C*. *elegans*.

### 2.3 Metformin increases healthspan via H3K4me3 modifiers because of reduced SAM levels

**FIGURE 3:**
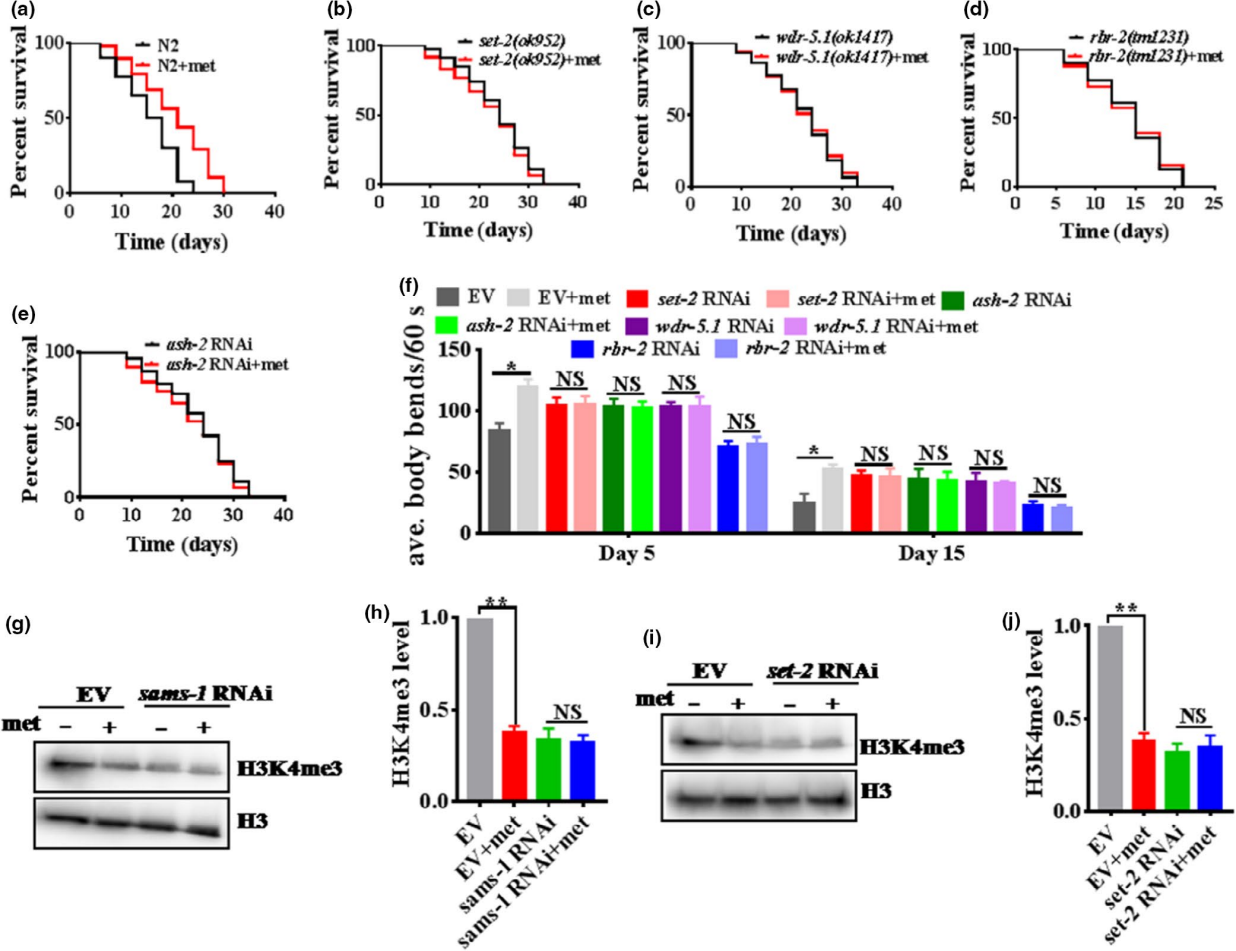
Metformin increases healthspan via H3K4me3 modifiers because of reduced SAM levels in adult worms. (a) Metformin (50 mM) enhances the longevity of WT worms (N2)(adults). (b–d) metformin failed to increase the lifespan of the H3K4me3 methyltransferases *set*‐*2(ok952)*, and *wdr*‐*5*.*1(ok1417)* mutants and H3K4me3 demethylase *rbr*‐*2(tm1231)* mutants, compared to that of WT worms (adults). (e) Similar results were also observed in the H3K4me3 methyltransferase *ash*‐*2* RNAi worms (adults). *p < 0.05 versus N2 or EV (empty vector) (log‐rank test). (f) Metformin treatment failed to increase the locomotory ability (determined by the average bends of the worm body per 60 s) of the H3K4me3 methyltransferases *set*‐*2*, *ash*‐*2*, and *wdr*‐*5*.*1* RNAi worms and the H3K4me3 demethylase *rbr*‐*2* RNAi worms (adults). These results are presented as the mean ± SD of three independent experiments performed in triplicate. *p < 0.05 versus EV (empty vector) (unpaired *t*‐test). NS, no significance. (g) Knockdown of *sams*‐*1* reduced the protein levels of H3K4me3 in *C*. *elegans*. Interestingly, metformin did not further decrease the H3K4me3 level in *sams*‐*1* RNAi worms (adults). (h) The right panel shows quantification of H3K4me3 intensity. (i) Metformin failed to reduce the H3K4me3 level in *set*‐*2* RNAi worms (adults). (j) The right panel shows quantification of H3K4me3 intensity. These results are presented as the mean ± SD of three independent experiments performed in triplicate. *p < 0.05 versus EV (empty vector) (unpaired *t*‐test). NS, no significance

Epigenetic alterations, including histone modification and chromatin remodeling, have been described as one of the hallmarks of aging (Han & Brunet, 2012). Previous studies have reported that genetic manipulations of histone modifiers can change specific histone mark levels and in turn affect the lifespan of individuals, such as the H3K4me3 complex (Greer et al., 2010) and H3K27me3 complex (Jin et al., 2011; Maures et al., 2011). To determine whether metformin‐induced SAM restriction prolonged healthspan through H3K4me3, we performed a lifespan assay with *C*. *elegans*. *set*‐*2*, *wdr*‐*5*.*1*, *rbr*‐*2*, and *ash*‐*2* are H3K4me3 complex members and also state that *set*‐*2*, *wdr*‐*5*, and *ash*‐*2* are already long‐lived and *rbr*‐*2* is already short‐lived (Greer et al., 2010). We found that metformin failed to enhance the lifespan of H3K4me3 methyltransferases *set*‐*2(ok952)* and *wdr*‐*5*.*1(ok1417)* mutants or H3K4me3 demethylase *rbr*‐*2(tm1231)* mutants compared with WT worms (Figure 3a–d and Table S1). Similar results were also observed for the H3K4me3 methyltransferase *ash*‐*2* RNAi worms (Figure 3e and Table S1). However, metformin promoted the lifespan of H3K36me3 putative methyltransferase *mes*‐*4* RNAi worms (Rechtsteiner et al., 2010), H3K9me3 putative methyltransferase *set*‐*25* RNAi worms (Lev et al., 2017), and H3K27me3 putative demethylase *utx*‐*1* RNAi worms (Figure S1A‐D and Table S2). Furthermore, we exposed *set*‐*2* and *rbr*‐*2* RNAi nematodes to 50‐mM metformin as described above and found that the lifespan‐extending effects of metformin were abolished (Figure S1E,F and Table S2). Taken together, these results suggest that metformin acts on H3K4me3 modifiers to extend the lifespan of *C*. *elegans*. As they age, *C*. *elegans* exhibited muscle deterioration and locomotion rate decline (Chen et al., 2017). Metformin treatment failed to increase the locomotory ability (determined by the average bends of the worm body per 60 s) and decrease age pigments in the H3K4me3 methyltransferases *set*‐*2*, *ash*‐*2*, and *wdr*‐*5*.*1* RNAi worms and the H3K4me3 demethylase *rbr*‐*2* RNAi worms (Figure 3f and Figure S2), indicating that metformin improved fitness depending on the H3K4me3 modifiers in *C*. *elegans*. Furthermore, in *C*. *elegans*, SAM is synthesized by the SAM synthase SAMS‐1. As expected, knockdown of *sams*‐*1* reduced the protein levels of H3K4me3 in *C*. *elegans*, consistent with previous work (Ding et al., 2015). Interestingly, metformin did not further decrease the H3K4me3 level in *sams*‐*1* RNAi worms (Figure 3g,h), indicating that metformin‐induced SAM restriction influenced H3K4me3 modifiers in *C*. *elegans*. In addition, we found that metformin failed to reduce the H3K4me3 level in *set*‐*2* RNAi worms (Figure 3i,j). In conclusion, these findings indicate that metformin‐induced SAM restriction extends healthspan via H3K4me3 modifiers in *C*. *elegans*.

### 2.4 The H3K4me3 methyltransferase complex is necessary for an intact germline and requires the continuous production of mature eggs to extend healthspan after metformin treatment

**FIGURE 4:**
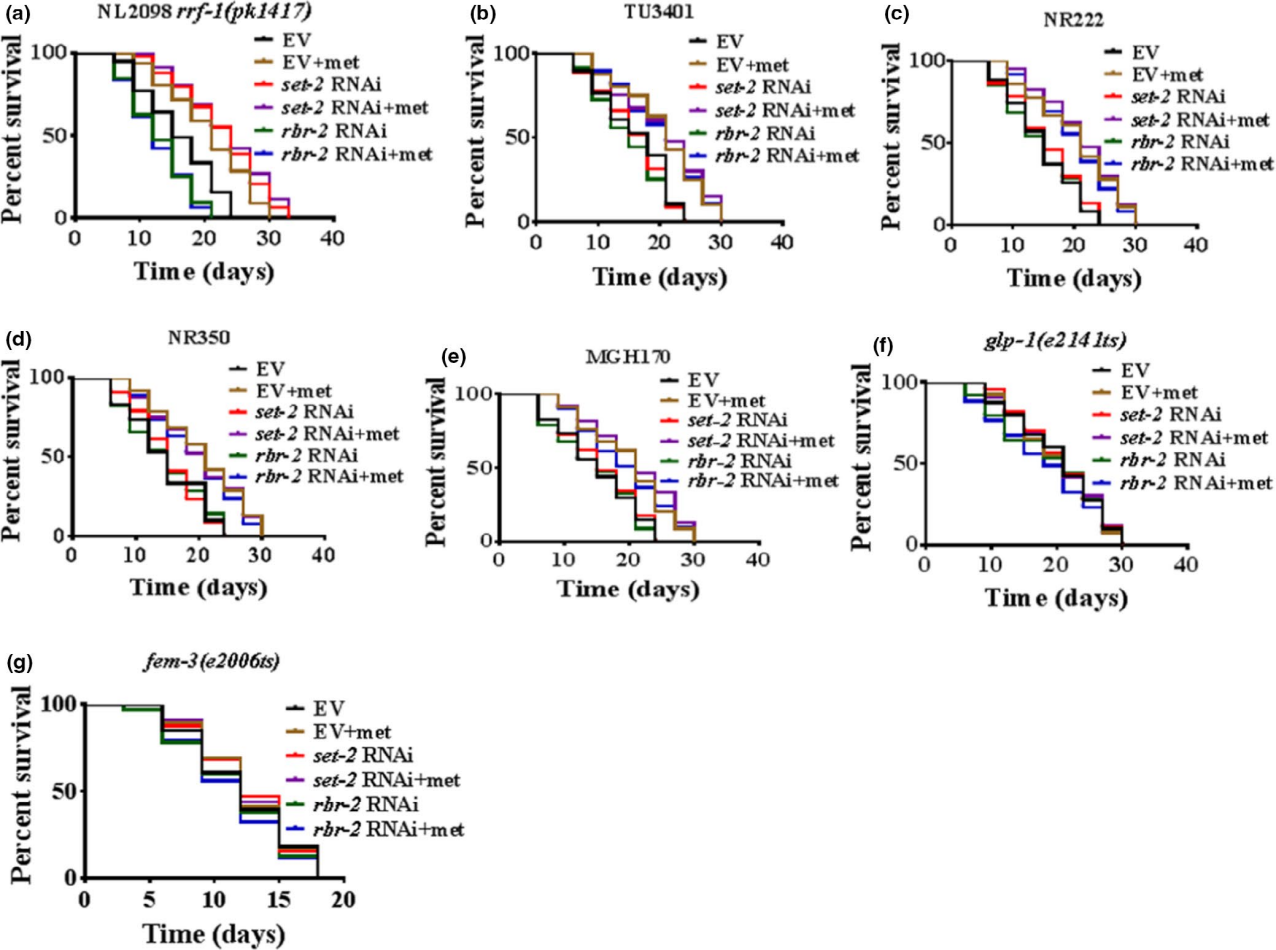
The H3K4me3 methyltransferase complex is necessary for an intact germline and requires the continuous production of mature eggs to extend healthspan after metformin treatment. (a) Metformin (50 mM) did not enhance the lifespan of *rrf*‐*1(pk1417)* knockdown *set*‐*2* and *rbr*‐*2* worms (adults). (b–e) RNAi of *set*‐*2* and *rbr*‐*2* in neurons, the hypodermis, muscle, and the intestine after metformin treatment promoted the lifespan of *C*. *elegans*. (f) Neither *set*‐*2* nor *rbr*‐*2* knockdown further extended the longevity of *glp*‐*1(e2141ts)* mutant worms (adults). (g) *set*‐*2* and *rbr*‐*2* knockdown did not extend the lifespan of *fem*‐*3(e2006ts)* mutant worms (adults). *p < 0.05 (log‐rank test)

To determine tissue‐specific actions of the H3K4me3 methyltransferase complex in response to healthspan after metformin treatment, we performed tissue‐specific gene knockdown experiments with the MGH170 strain in the intestine (Melo & Ruvkun, 2012), NR222 strain in the hypodermis (Qadota et al., 2007), NR350 strain in muscle (Qadota et al., 2007), TU3401 strain in neurons (Calixto et al., 2010), and NL2098 strain in the soma (such as the intestine, hypodermis) in addition to the germline (Kumsta & Hansen, 2012). We found that metformin treatment did increase the lifespan in empty vector‐treated NL2098 worms but failed to increase the lifespan in these worms when *set*‐*2* or *rbr*‐*2* were knocked down in the soma and germline (Figure 4a and Table S1). However, RNAi of *set*‐*2* and *rbr*‐*2* in neurons, the hypodermis, muscle, and the intestine after metformin treatment promoted the lifespan of *C*. *elegans* (Figure 4b–e and Table S2). In addition, to determine whether the presence of an intact germline was necessary for lifespan regulation by the H3K4me3 complex modifier after metformin treatment, we tested the effects of *set*‐*2* and *rbr*‐*2* knockdown in *glp*‐*1(e2141ts)* mutant worms, which developed only 5–15 meiotic germ cells not 1,500 germ cells, when exposed to a growth‐restrictive temperature at the L1 stage (Greer et al., 2010). As in Greer et al, we found that knock down of *set*‐*2* or *rbr*‐*2* in the germlineless worms failed to have any effect on lifespan (Greer et al., 2010). Additionally, we found that metformin had no effect on lifespan when the germline was deleted in the presence or absence of *set*‐*2* and *rbr*‐*2* (Figure 4f and Table S1), indicating that the presence of an intact germline was necessary for lifespan regulation by H3K4me3 modifiers after metformin treatment. A previous study reported that the H3K4 trimethylation complex regulated the lifespan in adult worms by a mechanism that depended on the presence of adult germline stem cells and/or the continuous production of eggs (Greer et al., 2010). Furthermore, to determine whether germline stem cells or the continuous production of eggs was necessary for the regulation of lifespan by H3K4me3 modifiers after metformin treatment, we used *fem*‐*3(e2006ts)* mutant worms, which had germline stem cells but did not produce fertilized eggs (Greer et al., 2010). *set*‐*2* and *rbr*‐*2* knockdown did not extend the lifespan of *fem*‐*3(e2006ts)* mutant worms (Figure 4g and Table S1), suggesting that the production of mature eggs, not merely the presence of germline stem cells, was required for *set*‐*2* and *rbr*‐*2* knockdown to extend lifespan after metformin treatment. Taken together, these results suggest that after metformin treatment, the H3K4me3 methyltransferase complex is necessary for an intact germline to extend healthspan and requires the continuous production of mature eggs for longevity.

### 2.5 A mTOR/RSKS‐1‐dependent mechanism mediates the healthspan extension switching of H3K4me3 methyltransferase‐deficient worms after metformin treatment

**FIGURE 5:**
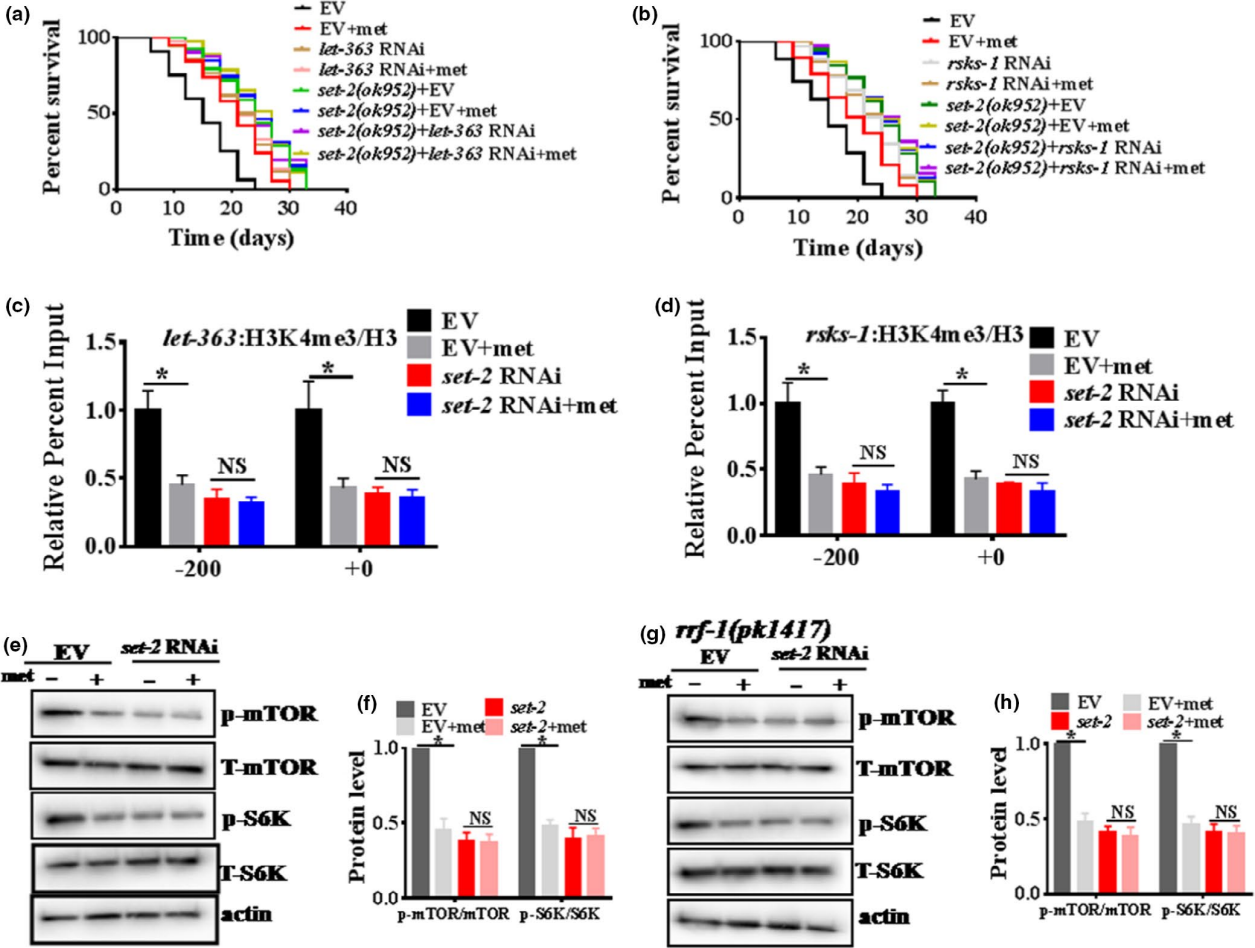
The mTOR/RSKS‐1 dependent machanism mediates the healthspan extension switching of H3K4me3 methyltransferase‐deficient worms after metformin treatment the adult *C*. *elegans*. (a, b) Knockdown of *let*‐*363*(a homologue of mammalian mTOR) or *rsks*‐*1*(a homologue of mammalian S6 kinase) did not further extend the lifespan of *set*‐*2(ok952)* mutant worms after metformin treatment (adults). *p < 0.05 (log‐rank test). (c, d) Chromatin immunoprecipitation comparing levels of H3K4me3 after metformin treatment in Empty Vector (EV) or *set*‐*2* RNAi worms. Input levels were normalized to the EV value on the upstream primer pair. Numerical representation of primer location is based on translational start site. *p < 0.05 versus EV (unpaired *t*‐test). (e–h) Metformin treatment significantly decreased the levels of mTOR and S6K phosphorylation. *set*‐*2* knockdown also significantly reduced the levels of mTOR and S6K phosphorylation. However, metformin treatment did not further decrease the levels of mTOR and S6K phosphorylation in the *set*‐*2* RNAi worms (adults). (f, h) The right panel shows quantification of p‐mTOR/mTOR, and p‐S6K/S6K. These results are presented as the mean ± SD of three independent experiments performed in triplicate. *p < 0.05 versus EV (unpaired *t*‐test). NS, no significance

Previous studies have shown that metformin inhibits the mTOR signaling pathway to enhance the longevity of *C*. *elegans* (Chen et al., 2017; Wu et al., 2016). In addition, a recent study reported that fat metabolism switching in H3K4me3 methyltransferase‐deficient worms mediated at least in part by the downregulation of germline targets, including mTOR/RSKS‐1 (Han et al., 2017). Whether mTOR/RSKS‐1 is downstream of the H3K4me3 methyltransferase complex and extends the healthspan of *C*. *elegans* after metformin treatment remains unclear. Notably, knockdown of *let*‐*363* (a homologue of mammalian mTOR) or *rsks*‐*1* (a homologue of mammalian S6 kinase) did not further extend lifespan in *set*‐*2(ok952)* mutant worms before or after metformin treatment (Figure 5a,b and Table S1). In addition, after metformin treatment, *let*‐*363* or *rsks*‐*1* knockdown failed to increase the locomotory ability (determined by the average bends of the worm body per 60 s) or decrease age pigments of the H3K4me3 methyltransferases *set*‐*2(ok952)* mutant worms (Figures S3A,B and S4A,B). In addition, to examine H3K4me3 levels on these specific candidate genes *let*‐*363* and *rsks*‐*1* before and after metformin treatment with or without *set*‐*2*, using chromatin immunoprecipitations with antibodies specific to H3K4me3, we found that metformin treatment decreased the H3K4me3 levels at *let*‐*363* and *rsks*‐*1* in empty vector‐treated worms but failed to decrease the H3K4me3 levels at these genes in *set*‐*2* RNAi worms (Figure 5c,d). Furthermore, our results found that metformin treatment significantly decreased the levels of mTOR and S6K phosphorylation (Figure 5e,f), suggesting inhibition of the TORC1 pathway, consistent with the previous work (Chen et al., 2017). Notably, *set*‐*2* knockdown also significantly reduced the levels of mTOR and S6K phosphorylation (Figure 5e,f). However, metformin treatment did not further decrease the levels of mTOR and S6K phosphorylation in *set*‐*2* RNAi worms (Figure 5e,f), indicating that mTOR/RSKS‐1 was downstream of the H3K4me3 modifiers to extend the healthspan of *C*. *elegans* after metformin treatment. In addition, knockdown of *set*‐*2* in *rrf*‐*1(pk1417)* worms also significantly reduced the levels of mTOR and S6K phosphorylation, and *let*‐*363* RNAi also reduced the levels of mTOR phosphorylation (Figure 5g,h, Figure S5). However, metformin treatment did not further decrease the level of mTOR or S6K phosphorylation in *rrf*‐*1(pk1417)* and *set*‐*2* RNAi worms (Figure 5g,h), suggesting that the H3K4me3 methyltransferase complex regulated the mTOR/RSKS‐1 signaling pathway after metformin treatment. Previous studies have shown that *aak*‐*2* (AMPK) (Cabreiro et al., 2013; Chen et al., 2017; Onken & Driscoll, 2010) and *skn*‐*1* (Onken & Driscoll, 2010) are classical targets of metformin that elicit longevity. To better understand whether metformin‐altered H3K4me3 modifiers regulated the *aak*‐*2* or *skn*‐*1* to extend the lifespan, we tested the level of AMPK phosphorylation and the expression of *gst*‐*4*::GFP, which is a target gene of *skn*‐*1*. Our results indicated that metformin treatment significantly increased the level of AMPK phosphorylation (Figure S6A and B), suggesting an activation of the AMPK pathway, consistent with the previous work (Cabreiro et al., 2013; Chen et al., 2017). Unexpectedly, *set*‐*2(ok952)* mutant worms did not affect the level of AMPK phosphorylation (Figure S6A,B). However, metformin treatment further increased the level of AMPK phosphorylation in *set*‐*2(ok952)* mutant worms (Figure S6A,B). Similar results were also confirmed for *gst*‐*4*::GFP (Figure S6C,D), indicating that metformin‐altered H3K4me3 modifiers did not depend on *aak*‐*2* or *skn*‐*1* to extend worm lifespan. Overall, these results suggest that the healthspan extension switch of H3K4me3 methyltransferase‐deficient worms after metformin treatment is mediated by downregulation of mTOR/RSKS‐1.

## 3 DISCUSSION

Cabreiro et al showed that the metformin‐induced SAM deficiency was completely dependent on the presence of live bacteria (Cabreiro et al., 2013). However, our results suggested that treating *C*. *elegans* with metformin caused a decrease in SAM levels and in the SAM/SAH ratio in adult worms. This may be due to using different metformin supplementation protocols. S‐Adenosylmethionine (SAM) is the sole methyl donor that modifiers histones, and its change affects histone and/or DNA methylation (Ding et al., 2015). Liu et al. found that the reduction in SAM via downregulation of *Sams* led to decreased levels of H3K4me3 and H3K9me2 in *Drosophila* S2 cells (Liu et al., 2015). In addition, threonine‐regulated SAM specifically affects H3K4me3 in mouse embryonic stem cells (Shyh‐Chang et al., 2013). Furthermore, in *C*. *elegans*, a decrease in SAM leads to a large decrease in global H3K4me3 levels (Ding et al., 2015). Additionally, a reduced SAMe/SAH ratio, as a measure of reduced methylation potential, can regulate lifespan via histone methylation. Notably, a full understanding of metformin‐induced SAM restriction is likely to require the identification of specific methyltransferases affected by changes in SAM and SAH. It is worth noting that previous studies have focused on a potential common mechanism underlying the action of metformin: DR (Cabreiro et al., 2013; Onken & Driscoll, 2010). Moreover, many studies have focused on inactivating specific methyltransferase complexes to research physiological roles of methylation (Ding et al., 2015). However, the decrease in SAM due to metformin may not impact all methyltransferases equivalently; therefore, it has been difficult to observe how changes in SAM levels interface with molecular mechanisms. Here, we used *C*. *elegans* to discern molecular mechanisms linking metformin‐induced SAM limitation to extend healthspan and histone methylation. Through genetic screening of *C*. *elegans*, metformin‐induced SAM restriction was found to extend the healthspan of *C*. *elegans* via H3K4me3 modifiers. Therefore, our results reveal the mechanisms connecting metformin‐induced SAM restriction to specific methyltransferases and molecular changes underlying healthspan.

Consistent with the previous work (De Haes et al., 2014), our results showed that treatment with metformin (50 mM) increased the lifespan of wild‐type adult worms but not larval worms. The precise mechanism by which this increase is achieved remains unknown. Notably, metformin (50 mM) treatment decreased SAM levels, increased SAH levels and decreased the SAM/SAH ratio in adult worms but not larval worms. Interestingly, metformin significantly decreased the levels of H3K4me3 not in larval worms but in adult worms. Furthermore, the observation that metformin‐triggered longevity requires the production of mature eggs may explain why the metformin‐promoted healthspan was not observed in earlier stages of *C*. *elegans*, in which the production of mature eggs was inhibited. As they age, *C*. *elegans* exhibit muscle deterioration and locomotion rate decline (Chen et al., 2017; Huang et al., 2004; Onken & Driscoll, 2010). Metformin treatment failed to increase the locomotory ability (determined by the average bends of the worm body per 60 s) and decrease age pigments of the H3K4me3 methyltransferases *set*‐*2*, *ash*‐*2*, and *wdr*‐*5*.*1* RNAi worms and H3K4me3 demethylase *rbr*‐*2* RNAi worms, indicating that metformin extended healthspan dependent on H3K4me3 modifiers in *C*. *elegans*.

The protein kinase mechanistic target of rapamycin (mTOR) signaling pathway senses and integrates a variety of environmental cues to regulate organismal growth and homeostasis (Laplante & Sabatini, 2012; Schmelzle & Hall, 2000). Previous studies have shown that metformin inhibits the mTOR signaling pathway to enhance the longevity of *C*. *elegans* (Chen et al., 2017; Wu et al., 2016). In addition, a recent study reported that fat metabolism switching in H3K4me3 methyltransferase‐deficient worms mediated at least in part by the downregulation of germline targets, including mTOR/RSKS‐1 (Han et al., 2017). Whether mTOR/RSKS‐1 is downstream of the H3K4me3 methyltransferase complex and extends the healthspan of *C*. *elegans* after metformin treatment remain unclear. Our results suggest that the healthspan extension switching in H3K4me3 methyltransferase‐deficient worms after metformin treatment is mediated by the downregulation of mTOR/RSKS‐1. Notably, important players in the metformin‐induced lifespan extension of *C*. *elegans* are the AMPK ortholog AMP‐activated kinase‐2 (AAK‐2) and the skinhead‐1 (SKN‐1) transcription factor (Cabreiro et al., 2013; Chen et al., 2017; Onken & Driscoll, 2010). However, in this study, our results revealed that metformin‐altered H3K4me3 modifiers did not depend on *aak*‐*2* or *skn*‐*1* to extend healthspan. Overall, the findings provide molecular links between meformin, SAM restriction, histone methylation, and healthspan and elucidate the mode action of metformin‐regulated healthspan extension, which will boost its therapeutic application in the treatment of human aging and age‐related diseases.

## 4 MATERIALS AND METHODS

### 4.1 Worm strains and cultivation

Worms were maintained and propagated under standard conditions as previously described (Brenner, 1974; Stiernagle, 2006). The following nematode strains were obtained from the Caenorhabditis Genetics Center (CGC), which was funded by the NIH Office of Research Infrastructure Programs (P40 OD010440): N2 Bristol wild‐type, CB4037 *glp*‐*1(e2141)*, NL2098 *rrf*‐*1(pk1417)*, CB3844 *fem*‐*3(e2006)*, RB1025 *set*‐*2(ok952)*, RB1304 *wdr*‐*5*.*1(ok1417)*, ZR1 *rbr*‐*2(tm1231)*, CL2166 N2 dvIs19[pAF15(*gst*‐*4*::GFP::NLS)] NR222 *rde*‐*1(ne219)*;kzIs9, NR350 *rde*‐*1(ne219)*;kzIs20, and TU3401 (*sid*‐*1(pk3321)*V;uIs69V) for neuronal‐specific RNAi. The nematode strain for intestinal‐specific RNAi MGH170 *(sid*‐*1(qt9)*, Is [*vha*‐*6pr*::*sid*‐*1*], and Is [*sur*‐*5pr*::*GFPNLS*] was kindly provided by Dr. Gary Ruvkun (Massachusetts General Hospital, Harvard Medical School, Boston, MA). *C*. *elegans* mutants were backcrossed three times with the WT strain (N2) used in the laboratory.

### 4.2 RNA Interference

The strains of *E*.*coli* used for RNAi were obtained from the Ahringer library (Kamath & Ahringer, 2003). RNAi feeding experiments were performed on synchronized L1 to L2 larvae at 20°C. Briefly, *E*. *coli* strain HT115(DE3)‐expressing dsRNA was grown overnight in LB broth containing 100‐μg/ml ampicillin at 37°C, and then spread on NGM plates containing 100‐μg/ml ampicillin and 5‐mM isopropyl 1‐thio‐β‐D‐galactopyranoside (IPTG). The RNAi‐expressing bacteria were grown overnight at 25°C. Synchronized L1 to L2 larvae were placed on RNAi plates until they reached maturity at 20°C. *Unc*‐*22* RNAi was included as a positive control in all experiments to account for RNAi efficiency.

### 4.3 Analysis of S‐adenosylmethionine and S‐adenosylhomocysteine

Quantification of SAMe and SAH was performed as described previously (Burren et al., 2006). Nematodes were synchronized and treated for 1 day with or without 50‐mM metformin starting at L1/L2 or L4 larvae stage.

### 4.4 Lifespan assays

For larvae lifespan assays: synchronized L1 to L2 larvae were placed on NGM plates with or without 50‐mM metformin at 20°C. For adults' lifespan assays: synchronized L1 to L2 larvae were placed on NGM plates until they reached maturity at 20°C. Approximately 100 L4‐stage (day 0 for the lifespan assays) worms (except for *glp*‐*1*) were transferred to NGM agar plates with or without 50‐mM metformin. The *glp*‐*1* mutant worms were cultured at 25°C until day 1 adult (day 0 for the lifespan assays), and ~100 worms were transferred to 20°C for the lifespan assay. For each experiment, except where otherwise noted, the lifespan of 100 animals was measured in each trial. For the lifespan experiments, we placed 15 L4‐stage larvae on 3 plates with 5 animals per plate and allowed them to develop to adulthood and then lay eggs over 24 h. These parental animals were then removed from the plates. Forty‐eight hours later, 100 (day 2) L4 larvae were transferred to fresh plates. These animals were transferred to fresh plates every day during the progeny production period and then every other day thereafter. Animals that did not move when gently prodded were scored as dead. Animals that crawled off the plate or died from vulva bursting were censored (Chen et al., 2017; De Haes et al., 2014; Onken & Driscoll, 2010).

### 4.5 Locomotion assays

Nematodes were synchronized and treated with or without 50‐mM metformin starting at L4 larvae stage. On days 5 and 15 of life, 30 individuals from the control and experimental plates were measured for body bend rate in liquid. Briefly, worms were placed in 20‐μl M9 buffer on a glass slide and filmed with a Zeiss Imager M2 microscope. Body bends were counted by reviewing each frame of the 60‐s film (Onken & Driscoll, 2010).

### 4.6 Age pigment fluorescence detection

Nematodes were synchronized and treated for 10 days with or without 50‐mM metformin starting at L4 larvae stage. Worms were mounted onto an agarose pad attached to a glass slide and photographed with Zeiss Imager M2 microscope. Fluorescence intensity was measured by Image J (Chen et al., 2017).

### 4.7 Fluorescence microscopy

Synchronized L1 worms of the GST‐4::GFP strain were transferred to agar plates supplemented with or without 50‐mM metformin. L4 worms were picked and placed in a drop of 0.06% levamisole and observed under a 10x objective. Images were obtained by using a Zeiss Axioskop 2 plus fluorescence microscope (Carl Zeiss, Jena, Germany) with a digital camera. The fluorescence intensity was quantified with the Image J software (NIH). Three plates with approximately 40 animals per plate were tested per assay, and all experiments were performed three times independently.

### 4.8 Western blotting

Nematodes were synchronized and treated for 1 day with or without 50‐mM metformin starting at L1/L2 or L4 larvae stage. After worms were homogenized in liquid nitrogen, the homogenate was lysed on ice for 60 min in lysis buffer (BioTeKe). Total proteins were loaded (40 μg per well) and separated on a 10% SDS polyacrylamide gel. Proteins were then transferred to Immobilon‐PSQ transfer PVDF membranes (Millipore, Bedford, MA). Primary antibodies against H3K4me3 (1:1,000 dilution; Abcam, ab8580), H3K9me3 (1:1,000 dilution; Abcam, ab8898), H3K27me3 (1:1,000 dilution; Abcam, ab272165), H3K36me3 (1:1,000 dilution; Abcam, ab9050), H3(1:2,000 dilution; Abcam, ab1791), p‐AMPKa (1:1,000 dilution, CST, 2535), p‐S6K (1:1,000 dilution, CST, 9205), S6K(1:1,000 dilution, CST, 2708), p‐mTOR (1:1,000 dilution, CST, 2971), mTOR (1:1,000 dilution, CST, 2972), and beta‐actin (1:1,000 dilution; Abcam, ab227387) used. The secondary antibody was a peroxidase‐coupled anti‐rabbit IgG (1:20,000 dilution; Abmart). The blots were developed using SuperSignal chemiluminescence substrate (Pierce). Band intensities were measured using ImageJ software.

### 4.9 Chromatin immunoprecipitation

Nematodes were synchronized and treated for 1 day with or without 50‐mM metformin starting at L4 larvae stage. L4/young adult *C*. *elegans* were collected and homogenized in liquid nitrogen. The homogenate was lysed on ice for 60 min in lysis buffer (250‐mM sucrose, 10‐mM KCl, 1.5‐mM MgCl2, 1‐mM EGTA, 1‐mM DTT, plus complete protease inhibitors), and then, nuclei were separated by centrifugation. Cross‐linking reactions, performed in 1% formaldehyde, were stopped by 100‐mM glycine before sonication. Ten micrograms of lysate was precleared with Protein A/G Dynabeads (Invitrogen), and then, incubated with antibodies to H3K4me3 (Abcam, ab8580) or Histone 3 (Abcam, ab1791). Immune complexes were precipitated with Protein A/G (Invitrogen). For quantitative PCR, a standard curve for each primer pair was used to determine the amount of DNA/Ct value and percent of input was determined and used for comparison between samples (Ding et al., 2015). The following primers were used for this study:

*let*‐*363*‐up‐primers:

*let*‐*363*‐F: GTCTATTATCGAACTATCA
*let*‐*363*‐R: GATTTAGTGCATTGTGGGC

*let*‐*363*‐TTS‐primers:

*let*‐*363*‐F: ACACAGGCGAACTAATACCGT
*let*‐*363*‐R: AGAGGCGGAAGAACAAGATGA

*rsks*‐*1*‐up‐primers:

*rsks*‐*1*‐F: TTCTTGCAGGTTATACGAAC
*rsks*‐*1*‐R: GGAGCAGCACGGAGTGGTG

*rsks*‐*1*‐TTS‐primers:

*rsks*‐*1*‐F: TTCACCTATGTCGCACCGTC
*rsks*‐*1*‐R: AGACGAAGCCAAATGTGCCAC

### 4.10 Statistical analysis

All western blotting quantifications were conducted in Image J. Statistical analyses for all data except for lifespan assays were carried out using the Student's *t*‐test (unpaired, two‐tailed) or ANOVA after testing for equal distribution of the data and equal variances within the data set. Survival data were analyzed by using the log‐rank (Mantel‐Cox) test. *p* < 0.05 was considered significant. The data were analyzed using the SPSS 17.0 software (IBM, Armonk, New York).

## CONFLICT OF INTEREST

None declared.

## AUTHOR CONTRIBUTIONS

Yun Liu, Yi Xiao, and Fang Liu conceptualized and designed the study, aided in acquiring and analyzing data, drafted, and critically revised the manuscript. Yi Xiao, Fang Liu, Qinghong Kong, XintingZhu, Haijuan Wang, SanhuaLi, NianJiang, and ChangyanYu participated in experiments and the data analysis. Yi Xiao wrote the paper. All authors read and approved the final manuscript.

## Supporting information

Fig S1‐S6

Table S1‐S2

## Data Availability

The data that support the findings of this study are available from the corresponding author upon reasonable request.
