# Supplementary material for: Metformin induces S‐adenosylmethionine restriction to extend the Caenorhabditis elegans healthspan through H3K4me3 modifiers
Source: Aging Cell. 2022 Feb 11;21(3):e13567. doi: 10.1111/acel.13567 (PMC8920454; doi:10.1111/acel.13567)
Supplement: Supplementary file 1 — Fig S1‐S6 [file ACEL-21-e13567-s002.docx]

**
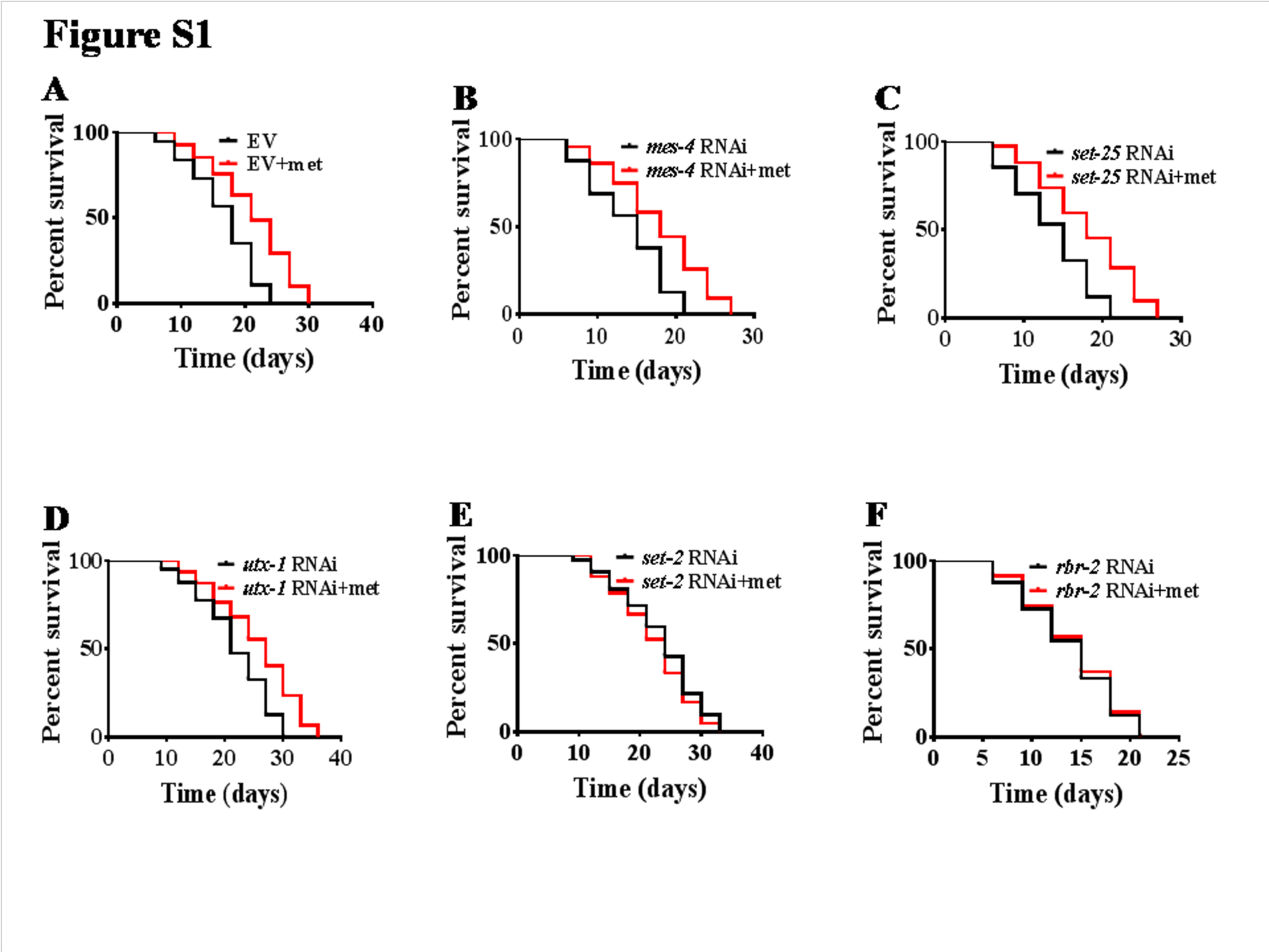
**

**Figure S1 (A-D) Metformin promoted the lifespan of H3K36me3 methyltransferase *mes-4* RNAi worms, H3K9me3 methyltransferase *set-25* RNAi worms and H3K27me3 demethylase *utx-1* RNAi worms. (E, F) Metformin failed to enhance the lifespan in *set-2* and *rbr-2* RNAi nematodes.** *P*< 0.05(log-rank test), relative to EV (empty vector).


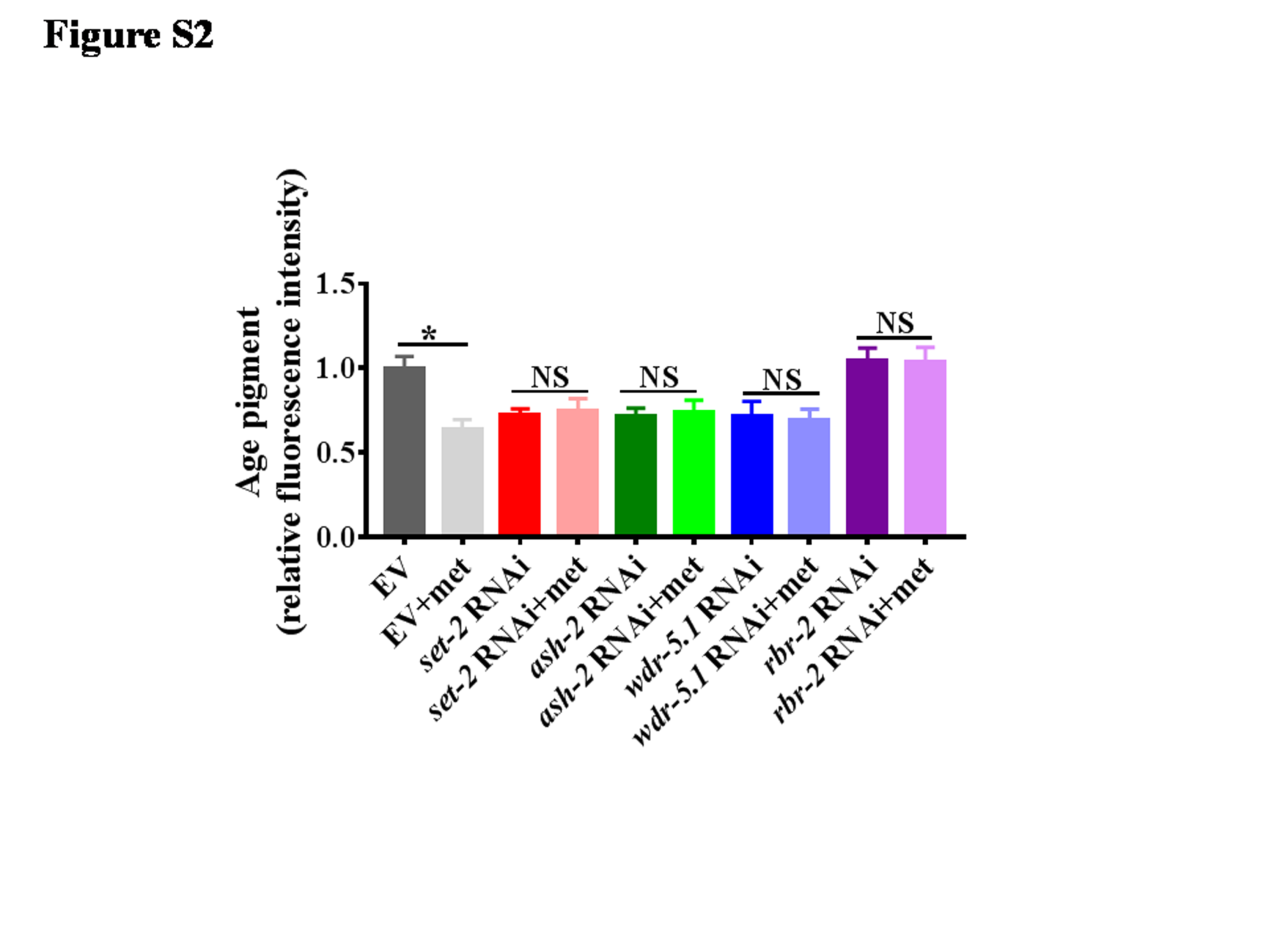


**Figure S2 Metformin treatment failed to decrease age pigments of the H3K4me3 methyltransferases *set-2*, *ash-2*, and *wdr-5.1* RNAi worms or the H3K4me3 demethylase *rbr-2* RNAi worms.** These results are mean ± SD of three independent experiments performed in triplicate. **P*< 0.05 versus EV (unpaired t-test). NS, no significance.


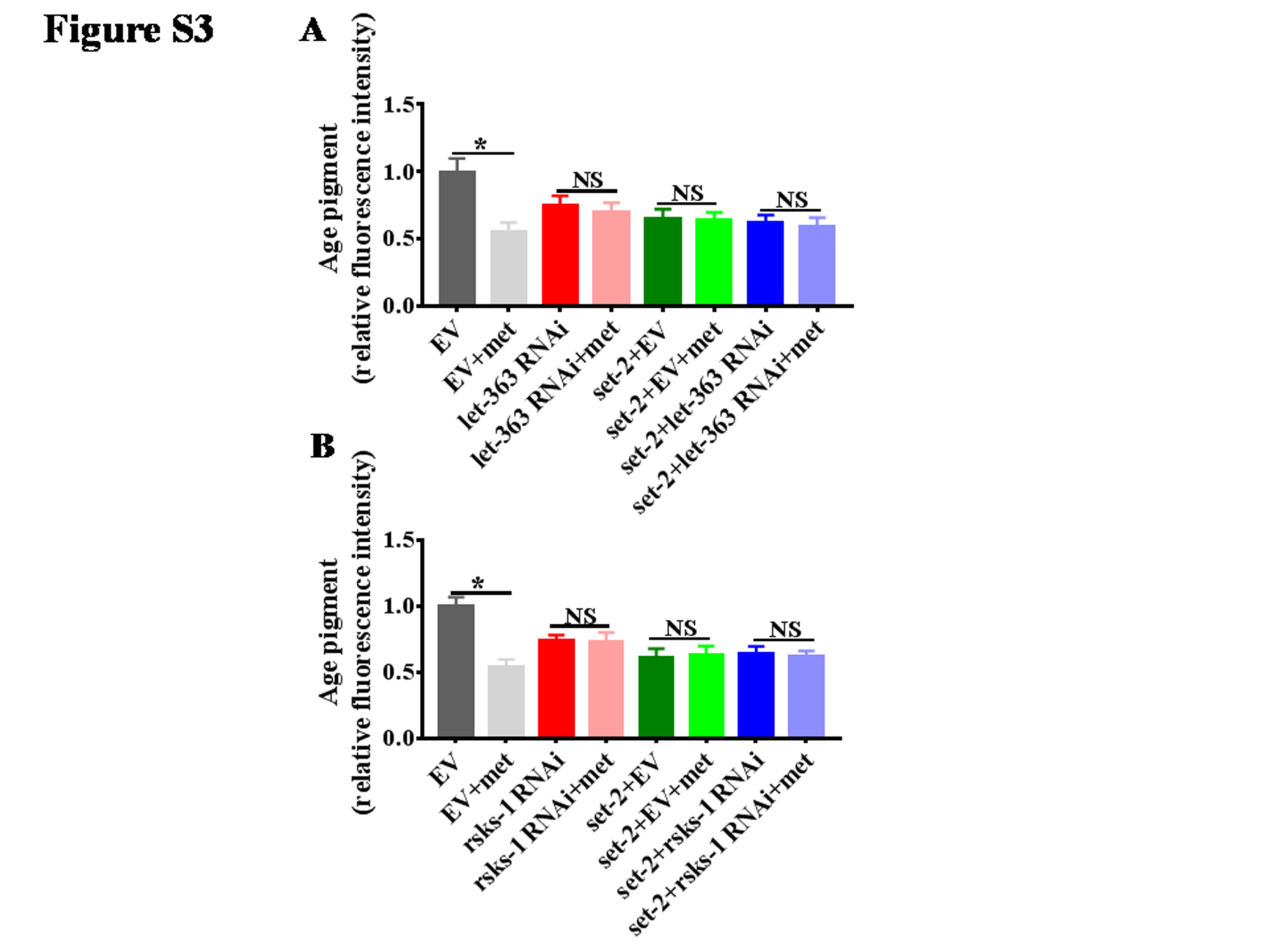


**Figure S3 (A, B) After metformin treatment, *let-363* or *rsks-1* knockdown failed to decrease age pigments of the H3K4me3 methyltransferase *set-2(ok952)* mutant worms.** These results are mean ± SD of three independent experiments performed in triplicate. **P*< 0.05 versus EV (unpaired t-test). NS, no significance.


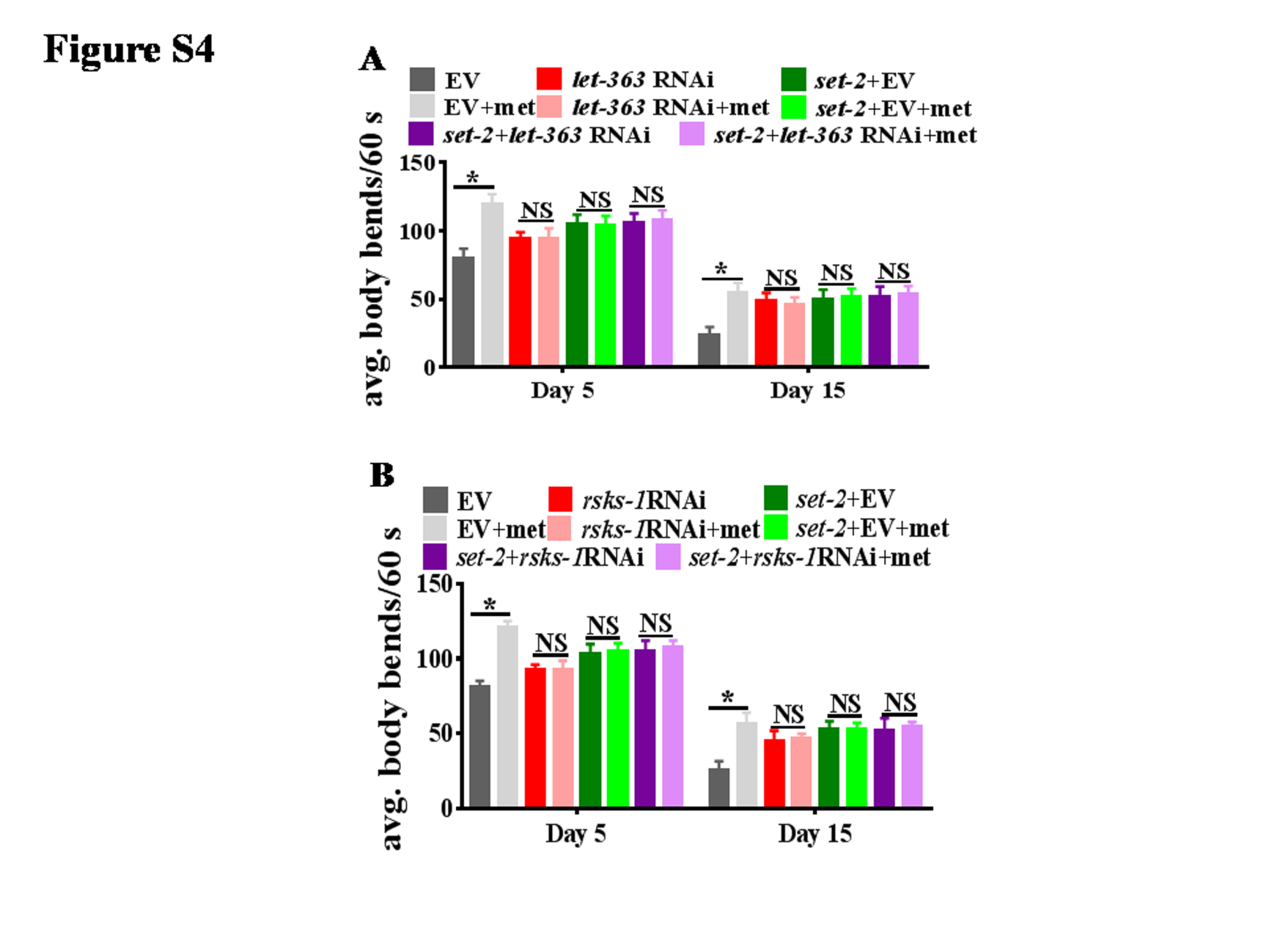


**Figure S4 (A, B) After metformin treatment, neither *let-363* nor *rsks-1* knockdown increased the locomotory ability (determined by the average bends of the worm body per 60 s) of the H3K4me3 methyltransferase *set-2(ok952)* mutant worms (adults).** These results are mean ± SD of three independent experiments performed in triplicate. **P*< 0.05 versus EV (unpaired t-test). NS, no significance.

**
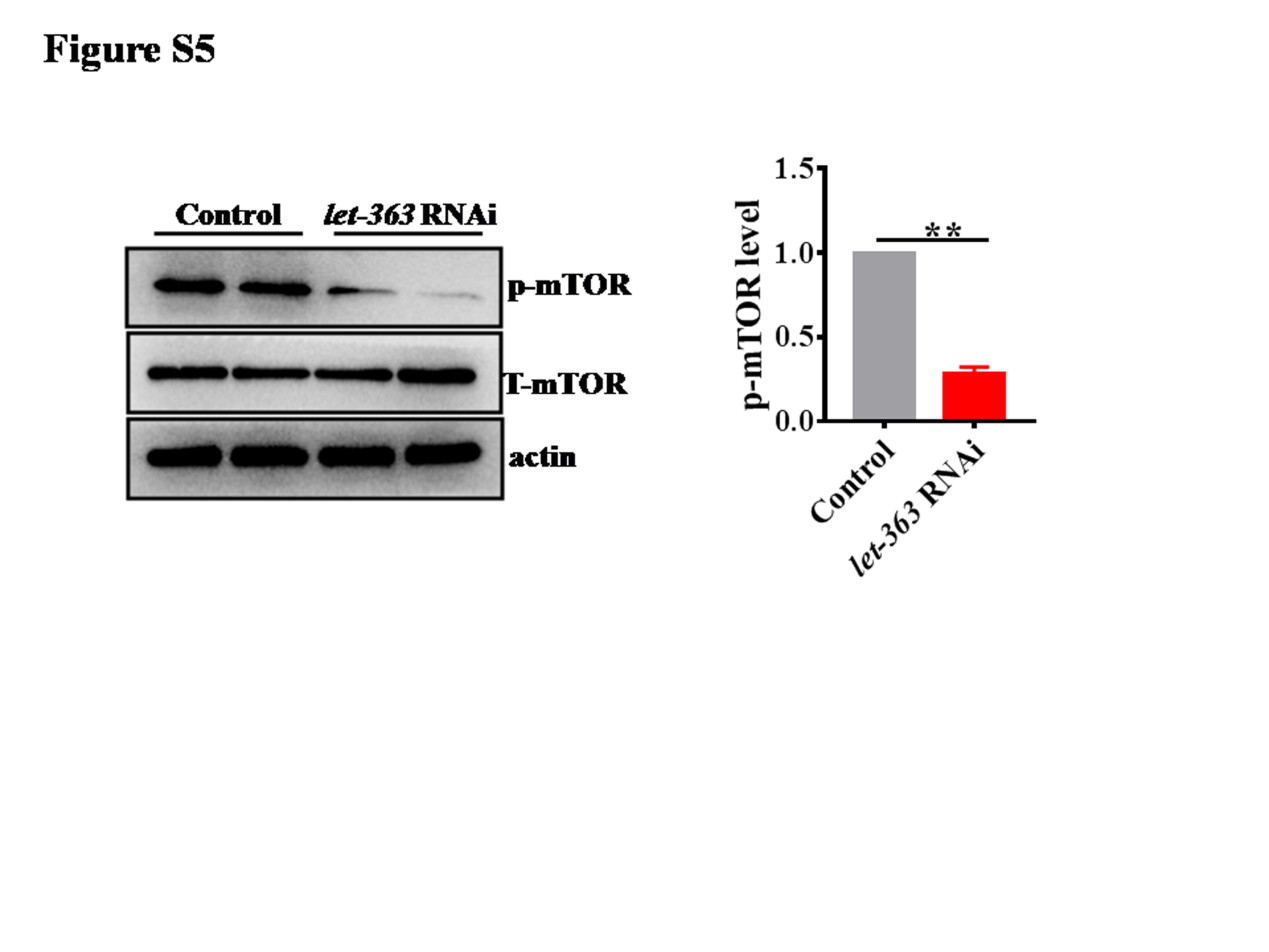
**

**Figure S5 *let-363* RNAi also reduced the levels of mTOR phosphorylation. The right panel shows quantification of p-mTOR intensity.** These results are mean ± SD of three independent experiments performed in triplicate. *P*< 0.05 (unpaired t-test).


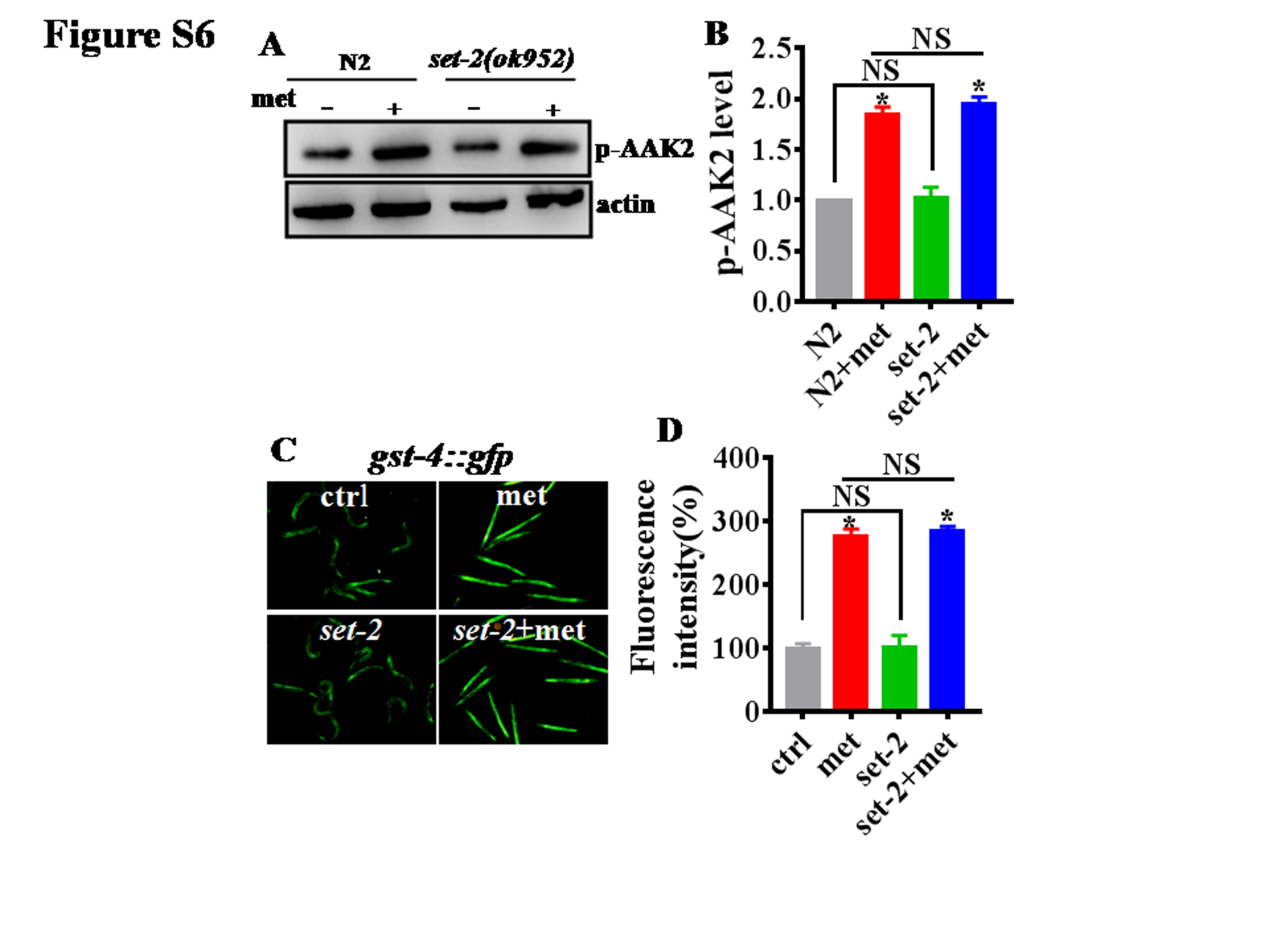


**Figure S6 (A) Metformin treatment significantly increased the levels of AMPK phosphorylation. Unexpectedly, *set-2(ok952)* mutant worms did not affect the levels of AMPK phosphorylation. However, metformin treatment could further increase the levels of AMPK phosphorylation in *set-2(ok952)* mutant worms. (B) The right panel shows quantification of p-AAK-2 intensity. (C) Similar results also confirmed in *gst-4*::GFP. (D) The right panel shows quantification of *gst-4*::GFP fluorescence intensity.** These results are mean ± SD of three independent experiments performed in triplicate. **P*< 0.05 versus control or N2 and *set-2* (wild-type worms) (unpaired t-test). NS, no significance.
