# Supplementary material for: Metformin induces S‐adenosylmethionine restriction to extend the Caenorhabditis elegans healthspan through H3K4me3 modifiers
Source: Aging Cell. 2022 Feb 11;21(3):e13567. doi: 10.1111/acel.13567 (PMC8920454; doi:10.1111/acel.13567)
Supplement: Supplementary file 2 — Table S1‐S2 [file ACEL-21-e13567-s001.doc]

**Supplementary Table 1. related to Figure 1, Figure 3, Figure 4, Figure 5.**

| **Strain/ Replicate Mean lifespan Worms Max lifespan p value**  **Treatment** **±SEM (days) Censored/Total ±SEM (days)** |
| --- |
| **N2(adult) 1(Graphed 16.82±0.34 9/72 23.65±0.46** |

**in Fig. 1A)**

**N2+50 mM 21.24±0.98 10/78 29.28±0.49 p＜0.05**

**metformin**

| **N2 2 17.12±0.36 12/92 24.15±0.45** |
| --- |

**N2+50 mM 21.75±0.39 11/90 30.68±0.46 p＜0.05**

**metformin**

| **N2 3 16.41±0.22 12/87 23.87±0.48** |
| --- |

**N2+50 mM 20.98±0.58 13/93 29.45±0.51 p＜0.05**

**metformin**

| **N2(larval) 1(Graphed 15.87±0.37 13/85 23.69±0.26** |
| --- |

**in Fig. 1B)**

**N2+50 mM 15.74±0.45 11/79 24.18±0.22 p=0.225**

**metformin**

| **N2 2 16.01±0.18 9/78 23.48±0.54** |
| --- |

**N2+50 mM 15.95±0.32 12/86 23.69±0.32 p=0.216**

**metformin**

| **N2 3 15.38±0.41 10/85 23.87±0.43** |
| --- |

**N2+50 mM 15.57±0.28 14/95 24.05±0.21 p=0.351**

**metformin**

| **N2 1(Graphed 16.57±0.39 15/95 23.73±0.29** |
| --- |

**in Fig. 3A)**

**N2+50 mM 20.65±0.42 11/75 29.58±0.12** **p＜0.05**

**metformin**

| **N2 2 16.81±0.37 10/80 23.79±0.24** |
| --- |

**N2+50 mM 21.46±0.36 12/88 30.39±0.37 p＜0.05**

**metformin**

| **N2 3 16.51±0.61 11/82 23.54±0.23** |
| --- |

**N2+50 mM 20.87±0.24 15/97 29.82±0.35 p＜0.05**

**metformin**

| ***set-2* 1(Graphed 24.18±0.22 8/78 33.48±0.42** |
| --- |

**in Fig. 3B)**

***set-2*+ 24.65±0.49 10/89 33.62±0.38 p=0.145**

**metformin**

| ***set-2* 2 24.76±0.41 8/72 33.76±0.39** |
| --- |

***set-2*+ 24.36±0.56 13/98 33.13±0.62 p=0.242**

**metformin**

| ***set-2* 3 23.79±0.69 7/76 33.72±0.34** |
| --- |

***set-2*+ 24.34±0.27 6/79 34.21±0.15 p=0.322**

**metformin**

| ***wdr-5.1* 1(Graphed 23.29±0.35 7/80 32.75±0.39** |
| --- |

**in Fig. 3C)**

***wdr-5.1*+ 23.78±0.62 9/79 32.76±0.46 p=0.214**

**metformin**

| ***wdr-5.1* 2 24.35±0.46 11/88 33.24±0.52** |
| --- |

***wdr-5.1*+ 24.72±0.39 12/92 33.36±0.26 p=0.325**

**metformin**

| ***wdr-5.1* 3 24.34±0.42 14/98 33.57±0.43** |
| --- |

***wdr-5.1*+ 24.12±0.46 12/90 33.12±0.84 p=0.218**

**metformin**

| ***rbr-2* 1(Graphed 14.78±0.64 11/89 21.36±0.53** |
| --- |

**in Fig. 3D)**

***rbr-2*+ 15.13±0.24 8/72 21.76±0.38 p=0.134**

**metformin**

| ***rbr-2* 2 15.37±0.25 12/91 20.78±0.37** |
| --- |

***rbr-2*+ 15.98±0.21 10/85 20.75±0.52 p=0.178**

**metformin**

| ***rbr-2* 3 14.86±0.49 9/89 20.92±0.67** |
| --- |

***rbr-2*+ 15.32±0.25 14/98 21.22±0.19 p=0.265**

**metformin**

| ***ash-2* RNAi 1(Graphed 24.55±0.29 8/86 33.42±0.36** |
| --- |

**in Fig. 3E)**

***ash-2* RNAi 24.14±0.54 7/83 33.29±0.25 p=0.156**

**+ metformin**

| ***ash-2* RNAi 2 23.28±0.79 11/89 32.78±0.74** |
| --- |

***ash-2*RNAi 24.08±0.16 9/78 33.21±0.51 p=0.241**

**+metformin**

| ***ash-2* RNAi 3 24.42±0.48 12/87 33.58±0.44** |
| --- |

***ash-2* RNAi 24.39±0.17 13/94 33.75±0.27 p=0.135**

**+metformin**

| ***rrf-1* 1(Graphed** |
| --- |

**EV in Fig. 4A) 15.32±0.38 10/87 23.74±0.65**

**EV+ 20.86±0.13 8/83 29.38±0.52 p＜0.05**

**metformin**

***set-2* RNAi 24.32±0.61 11/89 32.84±0.63**

***set-2* RNAi 24.75±0.83 13/87 33.16±0.47 p=0.232**

**+metformin**

***rbr-2* RNAi 13.53±0.65 7/89 20.54±0.62**

***rbr-2* RNAi 13.79±0.45 11/97 20.76±0.51 p=0.156**

**+metformin**

| ***rrf-1* 2** |
| --- |

**EV 16.13±0.32 8/73 24.26±0.41**

**EV+ 21.42±0.14 12/90 30.43±0.32 p＜0.05**

**metformin**

***set-2* RNAi 24.78±0.72 6/75 33.54±0.32**

***set-2* RNAi 24.25±0.18 10/83 33.19±0.28 p=0.176**

**+metformin**

***rbr-2* RNAi 13.59±0.62 7/79 21.14±0.61**

***rbr-2* RNAi 13.18±0.84 9/87 21.77±0.53 p=0.254**

**+metformin**

| ***rrf-1* 3** |
| --- |

**EV 16.58±0.71 10/79 23.97±0.68**

**EV+ 21.18±0.76 14/99 30.29±0.25 p＜0.05**

**metformin**

***set-2* RNAi 24.18±0.52 8/78 33.14±0.72**

***set-2* RNAi 23.91±0.67 7/98 33.19±0.13 p=0.431**

**+metformin**

***rbr-2* RNAi 13.12±0.62 7/89 21.34±0.53**

***rbr-2* RNAi 13.48±0.73 11/97 21.15±0.37 p=0.352**

**+metformin**

| ***glp-1* 1(Graphed** |
| --- |

**EV in Fig. 4B) 21.43±0.53 8/79 30.71±0.42**

**EV+ 20.94±0.38 11/91 30.31±0.27 p=0.356**

**metformin**

***set-2* RNAi 21.16±0.42 8/90 30.55±0.32**

***set-2* RNAi 21.32±0.64 7/78 29.48±0.38 p=0.443**

**+metformin**

***rbr-2* RNAi 21.19±0.56 10/89 29.84±0.43**

***rbr-2* RNAi 21.56±0.79 13/95 30.26±0.45 p=0.247**

**+metformin**

| ***glp-1* 2** |
| --- |

**EV 21.45±0.32 11/99 30.32±0.42**

**EV+ 21.78±0.26 13/91 29.46±0.71 p=0.255**

**metformin**

***set-2* RNAi 21.18±0.58 10/78 30.76±0.46**

***set-2* RNAi 20.71±0.77 8/88 30.12±0.35 p=0.337**

**+metformin**

***rbr-2* RNAi 20.72±0.37 11/89 30.47±0.76**

***rbr-2* RNAi 20.79±0.77 14/98 30.15±0.18 p=0.354**

**+metformin**

| ***glp-1* 3** |
| --- |

**EV 20.48±0.66 5/76 29.87±0.32**

**EV+ 20.81±0.57 10/85 29.43±0.64 p=0.315**

**metformin**

***set-2* RNAi 21.24±0.36 9/75 30.18±0.52**

***set-2* RNAi 21.51±0.78 7/70 30.45±0.23 p=0.368**

**+metformin**

***rbr-2* RNAi 20.88±0.76 6/70 30.17±0.58**

***rbr-2* RNAi 21.42±0.43 11/90 30.57±0.18 p=0.269**

**+metformin**

| ***fem-3* 1(Graphed** |
| --- |

**EV in Fig. 4C) 12.56±0.67 9/82 18.33±0.75**

**EV+ 12.79±0.22 13/89 18.26±0.37 p=0.274**

**metformin**

***set-2* RNAi 12.46±0.68 11/75 17.69±0.44**

***set-2* RNAi 12.11±0.47 9/70 18.45±0.36 p=0.285**

**+metformin**

***rbr-2* RNAi 12.81±0.32 11/78 18.17±0.38**

***rbr-2* RNAi 12.47±0.68 11/97 18.22±0.79 p=0.272**

**+metformin**

| ***fem-3* 2** |
| --- |

**EV 12.76±0.85 9/78 18.37±0.55**

**EV+ 12.56±0.32 10/86 18.45±0.34 p=0.332**

**metformin**

***set-2* RNAi 11.97±0.35 10/78 18.44±0.79**

***set-2* RNAi 12.31±0.69 7/80 18.48±0.45 p=0.351**

**+metformin**

***rbr-2* RNAi 12.85±0.37 8/76 18.12±0.77**

***rbr-2* RNAi 12.47±0.25 9/93 18.93±0.34 p=0.279**

**+metformin**

| ***fem-3* 3** |
| --- |

**EV 11.89±0.46 11/91 17.96±0.27**

**EV+ 11.77±0.98 13/96 17.65±0.34 p=0.169**

**metformin**

***set-2* RNAi 12.64±0.12 8/70 18.12±0.46**

***set-2* RNAi 11.86±0.24 7/76 18.48±0.22 p=0.338**

**+metformin**

***rbr-2* RNAi 12.56±0.91 11/90 18.22±0.79**

***rbr-2* RNAi 12.42±0.68 12/98 18.52±0.88 p=0.178**

**+metformin**

| **EV 1(Graphed 15.87±0.32 13/98 23.78±0.45** |
| --- |

**EV+ in Fig. 5A) 20.33±0.28 7/80 29.26±0.57 p＜0.05**

**metformin**

***let-363* 22.36±0.33 11/85 31.65±0.53**

***let-363*+ 22.47±0.25 10/88 30.74±0.45 p=0.427**

**metformin**

***set-2* 23.76±0.43 9/80 33.42±0.26**

***set-2* 23.89±0.14 7/86 33.58±0.17 p=0.314**

**+metformin**

***set-2*+*let-363* 24.75±0.25 11/87 33.18±0.46**

***set-2*+*let-363* 24.38±0.32 14/90 33.67±0.34 p=0.284**

**+metformin**

| **EV 2 16.27±0.33 13/98 24.58±0.45** |
| --- |

**EV+ 21.37±0.25 6/77 30.26±0.57 p＜0.05**

**metformin**

***let-363* 21.89±0.37 14/85 30.65±0.56**

***let-363*+ 21.73±0.29 12/79 30.38±0.15 p=0.276**

**metformin**

***set-2* 24.28±0.29 8/79 33.18±0.17**

***set-2* 24.81±0.38 7/90 33.46±0.39 p=0.344**

**+metformin**

***set-2*+*let-363* 24.87±0.28 11/78 33.26±0.17**

***set-2*+*let-363* 24.12±0.73 13/90 33.28±0.68 p=0.149**

**+metformin**

| **EV 3 16.52±0.31 13/89 23.58±0.42** |
| --- |

**EV+ 21.33±0.23 9/80 30.21±0.53 p＜0.05**

**metformin**

***let-363* 22.32±0.38 12/89 30.66±0.48**

***let-363*+ 22.68±0.45 14/92 30.51±0.19 p=0.332**

**metformin**

***set-2* 24.58±0.33 9/76 33.43±0.39**

***set-2* 24.78±0.37 7/80 33.49±0.54 p=0.315**

**+metformin**

***set-2*+*let-363* 24.55±0.89 13/90 33.78±0.48**

***set-2*+*let-363* 24.91±0.36 11/97 33.52±0.29 p=0.146**

**+metformin**

| **EV 1(Graphed 16.22±0.27 12/90 24.27±0.32** |
| --- |

**EV+ in Fig. 5B) 21.25±0.35 8/70 30.54±0.38 p＜0.05**

**metformin**

***rsks-1* 22.68±0.57 11/96 30.44±0.79**

***rsks-1*+ 22.93±0.42 9/85 30.69±0.37 p=0.521**

**metformin**

***set-2* 23.58±0.66 11/80 33.15±0.48**

***set-2* 23.48±0.87 14/86 33.87±0.12 p=0.368**

**+metformin**

***set-2*+*rsks-1* 24.14±0.58 10/85 33.28±0.32**

***set-2*+*rsks-1* 24.58±0.12 13/93 33.89±0.29 p=0.271**

**+metformin**

| **EV 2 15.88±0.76 11/92 24.34±0.11** |
| --- |

**EV+ 20.87±0.25 9/72 29.87±0.22 p＜0.05**

**metformin**

***rsks-1* 22.47±0.28 11/85 30.87±0.68**

***rsks-1*+ 22.65±0.34 14/99 30.27±0.52 p=0.189**

**metformin**

***set-2* 23.98±0.76 11/99 33.81±0.65**

***set-2* 24.26±0.58 14/95 33.32±0.69 p=0.366**

**+metformin**

***set-2*+*rsks-1* 24.33±0.21 13/79 33.98±0.43**

***set-2*+*rsks-1* 23.56±0.78 10/97 33.76±0.13 p=0.113**

**+metformin**

| **EV 3 16.82±0.65 11/82 24.92±0.22** |
| --- |

**EV+ 21.58±0.44 9/76 30.58±0.59 p＜0.05**

**metformin**

***rsks-1* 22.37±0.78 10/88 30.19±0.89**

***rsks-1*+ 22.89±0.95 11/90 30.23±0.75 p=0.472**

**metformin**

***set-2* 23.78±0.83 11/78 33.65±0.82**

***set-2* 24.36±0.75 14/87 33.46±0.61 p=0.387**

**+metformin**

***set-2*+*rsks-1* 23.68±0.79 12/91 33.21±0.15**

***set-2*+*rsks-1* 24.25±0.64 11/87 33.79±0.28 p=0.269**

**+metformin**

|  |
| --- |

**Supplementary Table 2. related to Figure S1, Figure 4(B-E)**

| **Strain/ Replicate Mean lifespan Worms Max lifespan p value**  **Treatment** **±SEM (days) Censored/Total ±SEM (days)** |
| --- |
| **EV 1(Graphed 15.45±0.23 14/99 24.28±0.35** |

**in Fig. S1A)**

**EV+ 21.52±0.53 12/89 30.54±0.27 p＜0.05**

**metformin**

| **EV 2 16.56±0.53 9/84 24.27±0.32** |
| --- |

**EV+ 21.48±0.26 13/79 30.38±0.55 p＜0.05**

**metformin**

| **EV 3 16.48±0.72 11/94 23.91±0.85** |
| --- |

**EV+ 21.78±0.46 9/90 30.35±0.64 p＜0.05**

**metformin**

| ***mes-4* RNAi 1(Graphed 15.36±0.49 13/91 21.53±0.42** |
| --- |

**in Fig. S1B)**

***mes-4* RNAi 18.49±0.58 10/89 27.17±0.35 p＜0.05**

**+metformin**

| ***mes-4* RNAi 2 15.79±0.59 10/75 20.75±0.56** |
| --- |

***mes-4* RNAi 18.68±0.51 11/79 27.73±0.21 p＜0.05**

**+metformin**

| ***mes-4* RNAi 3 16.25±0.19 13/91 21.47±0.49** |
| --- |

***mes-4* RNAi 18.89±0.36 10/89 27.68±0.25 p＜0.05**

**+metformin**

| ***set-25* RNAi 1(Graphed 16.11±0.35 13/98 21.48±0.58** |
| --- |

**in Fig. S1C)**

***set-25* RNAi 18.54±0.89 10/79 27.67±0.29 p＜0.05**

**+metformin**

| ***set-25* RNAi 2 15.87±0.32 14/91 21.69±0.22** |
| --- |

***set-25* RNAi 18.58±0.76 9/78 27.16±0.31 p＜0.05**

**+metformin**

| ***set-25* RNAi 3 15.72±0.58 11/78 21.18±0.97** |
| --- |

***set-25* RNAi 18.32±0.12 10/83 27.74±0.42 p＜0.05**

**+metformin**

| ***utx-1* RNAi 1(Graphed 21.54±0.42 14/94 30.37±0.27** |
| --- |

**in Fig. S1D)**

***utx-1* RNAi 26.88±0.38 10/86 35.79±0.87 p＜0.05**

**+metformin**

| ***utx-1* RNAi 2 21.62±0.66 9/74 30.69±0.16** |
| --- |

***utx-1* RNAi 27.51±0.27 11/89 36.27±0.24 p＜0.05**

**+metformin**

| ***utx-1* RNAi 3 21.79±0.83 14/89 30.45±0.74** |
| --- |

***utx-1* RNAi 27.36±0.62 9/79 36.52±0.71 p＜0.05**

**+metformin**

| ***set-2* RNAi 1(Graphed 24.51±0.89 14/90 33.43±0.42** |
| --- |

**in Fig. S1E)**

***set-2* RNAi 24.86±0.25 10/99 33.78±0.65 p=0.262**

**+metformin**

| ***set-2* RNAi 2 23.89±0.68 9/75 33.64±0.56** |
| --- |

***set-2* RNAi 24.57±0.31 11/85 33.19±0.12 p=0.173**

**+metformin**

| ***set-2* RNAi 3 24.11±0.79 15/95 33.69±0.41** |
| --- |

***set-2* RNAi 24.67±0.58 13/99 33.27±0.38 p=0.381**

**+metformin**

| ***rbr-2* RNAi 1(Graphed 15.25±0.39 10/82 21.76±0.41** |
| --- |

**in Fig. S1F)**

***rbr-2* RNAi 14.97±0.52 13/89 21.23±0.73 p=0.192**

**+metformin**

| ***rbr-2* RNAi 2 15.75±0.21 14/90 21.78±0.52** |
| --- |

***rbr-2*RNAi 15.38±0.28 11/88 21.12±0.67 p=0.428**

**+metformin**

| ***rbr-2* RNAi 3 15.28±0.19 9/77 21.73±0.55** |
| --- |

***rbr-2* RNAi 15.87±0.21 13/95 21.66±0.28 p=0.375**

**+metformin**

| **TU3401 1(Graphed** |
| --- |

**EV in Fig. 4B) 16.27±0.45 11/75 24.69±0.23**

**EV+ 21.32±0.56 12/74 30.33±0.41 p＜0.05**

**metformin**

***set-2* RNAi 15.87±0.46 8/89 23.72±0.67**

***set-2* RNAi 21.22±0.56 11/96 30.87±0.16 p＜0.05**

**+metformin**

***rbr-2* RNAi 16.57±0.48 8/85 24.22±0.17**

***rbr-2* RNAi 21.36±0.76 11/84 30.88±0.16 p＜0.05**

**+metformin**

| **TU3401 2** |
| --- |

**EV 16.57±0.27 12/78 24.61±0.22**

**EV+ 21.38±0.54 10/89 30.46±0.27 p＜0.05**

**metformin**

***set-2* RNAi 16.68±0.34 7/75 23.44±0.31**

***set-2* RNAi 21.55±0.17 9/72 30.17±0.36 p＜0.05**

**+metformin**

***rbr-2* RNAi 16.57±0.48 9/99 24.26±0.55**

***rbr-2* RNAi 21.21±0.76 11/82 30.82±0.18 p＜0.05**

**+metformin**

| **TU3401 3** |
| --- |

**EV 15.71±0.49 9/83 24.17±0.28**

**EV+ 21.36±0.28 13/93 30.27±0.48 p＜0.05**

**metformin**

***set-2* RNAi 15.98±0.45 7/87 24.45±0.84**

***set-2* RNAi 21.65±0.43 11/90 30.36±0.52 p＜0.05**

**+metformin**

***rbr-2* RNAi 16.57±0.44 8/79 24.28±0.87**

***rbr-2* RNAi 21.21±0.56 11/92 30.82±0.36 p＜0.05**

**+metformin**

| **NR222 1(Graphed** |
| --- |

**EV in Fig. 4C) 16.46±0.77 9/83 23.71±0.22**

**EV+ 21.24±0.24 11/91 30.38±0.25 p＜0.05**

**metformin**

***set-2* RNAi 17.11±0.12 13/96 24.79±0.51**

***set-2* RNAi 21.85±0.47 12/98 30.19±0.75 p＜0.05**

**+metformin**

***rbr-2* RNAi 15.98±0.46 8/80 24.65±0.34**

***rbr-2* RNAi 21.27±0.76 11/92 30.56±0.26 p＜0.05**

**+metformin**

| **NR222 2** |
| --- |

**EV 16.65±0.39 8/76 24.38±0.29**

**EV+ 21.58±0.46 13/80 30.16±0.21 p＜0.05**

**metformin**

***set-2* RNAi 16.34±0.79 11/93 24.69±0.57**

***set-2*RNAi 21.56±0.84 13/89 30.45±0.68 p＜0.05**

**+metformin**

***rbr-2* RNAi 16.57±0.45 12/89 24.22±0.67**

***rbr-2* RNAi 21.27±0.72 11/77 30.88±0.16 p＜0.05**

**+metformin**

| **NR222 3** |
| --- |

**EV 16.36±0.41 9/78 24.56±0.46**

**EV+ 21.27±0.55 10/86 30.78±0.14 p＜0.05**

**metformin**

***set-2* RNAi 16.58±0.38 9/78 24.47±0.34**

***set-2* RNAi 21.77±0.25 13/98 30.68±0.57 p＜0.05**

**+metformin**

***rbr-2* RNAi 16.25±0.41 8/75 24.27±0.87**

***rbr-2* RNAi 21.16±0.22 14/92 30.54±0.32 p＜0.05**

**+metformin**

| **NR350 1(Graphed** |
| --- |

**EV in Fig. 4D) 17.24±0.55 10/92 23.83±0.95**

**EV+ 20.79±0.29 13/86 29.16±0.78 p＜0.05**

**metformin**

***set-2* RNAi 17.21±0.24 9/96 24.26±0.16**

***set-2* RNAi 21.66±0.43 14/90 30.36±0.48 p＜0.05**

**+metformin**

***rbr-2* RNAi 16.87±0.42 12/99 24.55±0.87**

***rbr-2* RNAi 21.48±0.28 11/92 30.56±0.19 p＜0.05**

**+metformin**

| **NR350 2** |
| --- |

**EV 16.98±0.85 9/78 24.58±0.34**

**EV+ 21.72±0.16 10/96 30.76±0.44 p＜0.05**

**metformin**

***set-2* RNAi 16.86±0.21 11/89 24.36±0.81**

***set-2* RNAi 21.57±0.38 14/92 30.37±0.46 p＜0.05**

**+metformin**

***rbr-2* RNAi 17.24±0.57 12/89 24.27±0.39**

***rbr-2* RNAi 21.16±0.35 11/72 30.17±0.84 p＜0.05**

**+metformin**

| **NR350 3** |
| --- |

**EV 17.12±0.37 14/91 24.31±0.52**

**EV+ 21.47±0.62 13/96 30.44±0.94 p＜0.05**

**metformin**

***set-2* RNAi 17.34±0.26 12/98 24.46±0.29**

***set-2* RNAi 21.28±0.58 11/78 30.38±0.92 p＜0.05**

**+metformin**

***rbr-2* RNAi 16.75±0.38 9/99 24.35±0.54**

***rbr-2* RNAi 24.27±0.13 12/82 30.69±0.23 p＜0.05**

**+metformin**

| **MGH170 1(Graphed** |
| --- |

**EV in Fig. 4E) 16.55±0.78 15/97 24.78±0.57**

**EV+ 21.27±0.55 8/93 30.33±0.28 p＜0.05**

**metformin**

***set-2* RNAi 16.39±0.17 12/80 24.33±0.93**

***set-2* RNAi 21.89±0.81 11/87 30.59±0.51 p＜0.05**

**+metformin**

***rbr-2* RNAi 16.36±0.37 13/99 24.78±0.89**

***rbr-2* RNAi 21.26±0.86 11/89 30.66±0.56 p＜0.05**

**+metformin**

| **MGH170 2** |
| --- |

**EV 16.99±0.39 14/93 24.51±0.37**

**EV+ 21.22±0.83 12/88 30.15±0.35 p＜0.05**

**metformin**

***set-2* RNAi 16.73±0.39 8/97 24.78±0.92**

***set-2* RNAi 21.34±0.57 12/99 30.74±0.32 p＜0.05**

**+metformin**

***rbr-2* RNAi 16.31±0.45 11/73 24.27±0.16**

***rbr-2* RNAi 21.71±0.23 11/76 30.89±0.76 p＜0.05**

**+metformin**

| **MGH170 3** |
| --- |

**EV 16.87±0.84 12/87 24.29±0.37**

**EV+ 21.26±0.65 12/89 30.59±0.27 p＜0.05**

**metformin**

***set-2* RNAi 16.88±0.68 13/78 24.89±0.43**

***set-2* RNAi 21.44±0.79 11/98 30.57±0.79 p＜0.05**

**+metformin**

***rbr-2* RNAi 16.77±0.38 11/78 24.47±0.97**

***rbr-2* RNAi 21.93±0.27 12/79 30.43±0.53 p＜0.05**

**+metformin**

|  |
| --- |
